# Inferiority or Even Superiority of Virtual Reality Exposure Therapy in Phobias?—A Systematic Review and Quantitative Meta-Analysis on Randomized Controlled Trials Specifically Comparing the Efficacy of Virtual Reality Exposure to Gold Standard *in vivo* Exposure in Agoraphobia, Specific Phobia, and Social Phobia

**DOI:** 10.3389/fpsyg.2019.01758

**Published:** 2019-09-10

**Authors:** Theresa F. Wechsler, Franziska Kümpers, Andreas Mühlberger

**Affiliations:** Department for Clinical Psychology and Psychotherapy, Institute of Psychology, University of Regensburg, Regensburg, Germany

**Keywords:** anxiety disorder, agoraphobia, social anxiety, specific phobia, exposure therapy, virtual reality, meta-analysis, systematic review

## Abstract

**Background:** Convincing evidence on Virtual Reality (VR) exposure for phobic anxiety disorders has been reported, however, the benchmark and golden standard for phobia treatment is *in vivo* exposure. For direct treatment comparisons, the control of confounding variables is essential. Therefore, the comparison of VR and *in vivo* exposure in studies applying an equivalent amount of exposure in both treatments is necessary.

**Methods:** We conducted a systematic search of reports published until June 2019. Inclusion criteria covered the diagnosis of Specific Phobia, Social Phobia, or Agoraphobia, and a randomized-controlled design with an equivalent amount of exposure in VR and *in vivo*. We qualitatively reviewed participants' characteristics, materials, and the treatment procedures of all included studies. For quantitative synthesis, we calculated Hedges' *g* effect sizes for the treatment effects of VR exposure, *in vivo* exposure, and the comparison of VR to *in vivo* exposure in all studies and separately for studies on each diagnosis.

**Results:** Nine studies (*n* = 371) were included, four on Specific Phobia, three on Social Phobia, and two on Agoraphobia. VR and *in vivo* exposure both showed large, significant effect sizes. The comparison of VR to *in vivo* exposure revealed a small, but non-significant effect size favoring *in vivo* (*g* = −0.20). Specifically, effect sizes for Specific Phobia (*g* = −0.15) and Agoraphobia (*g* = −0.01) were non-significant, only for Social Phobia we found a significant effect size favoring *in vivo* (*g =* −*0.50*). Except for Agoraphobia, effect sizes varied across studies from favoring VR to favoring *in vivo* exposure.

**Conclusions:** We found no evidence that VR exposure is significantly less efficacious than *in vivo* exposure in Specific Phobia and Agoraphobia. The wide range of study specific effect sizes, especially in Social Phobia, indicates a high potential of VR, but also points to the need for a deeper investigation and empirical examination of relevant working mechanisms. In Social Phobia, a combination of VR exposure with cognitive interventions and the realization of virtual social interactions targeting central fears might be advantageous. Considering the advantages of VR exposure, its dissemination should be emphasized. Improvements in technology and procedures might even yield superior effects in the future.

## Introduction

### Rationale

#### Phobic Anxiety Disorders

Phobic anxiety disorders (ICD-10 F40) are listed as a subcategory of anxiety disorders in the ICD-10 (World Health Organization, 1992). They are characterized by anxiety in circumscribed situations, which currently pose little or no actual danger, and by an avoidance of those situations or an endurance with dread (World Health Organization, 1992). There are three subtypes of phobic anxiety disorders in the ICD-10 (World Health Organization, 1992): Agoraphobia (F40.0), Social Phobia (F40.1), and Specific Phobia (F40.2). Patients with Specific Phobia fear specific situations or objects such as animals, heights, thunder, darkness or closed spaces. Social Phobia patients report fear of scrutiny by other people, which leads to an avoidance of social situations. Agoraphobia is characterized by a fear of situations in which fleeing from the situation or help is not easily accessible, such as crowds in public spaces, leaving home, entering shops, or traveling alone in a train, bus or plane. It can be coded with (F40.01) or without (F40.00) Panic Disorder. Other anxiety disorders (ICD-10 F41) include Panic Disorder (F41.0), or generalized anxiety disorders (GAD) (F41.1) (World Health Organization, 1992). Both anxiety disorders are related to internal stimuli, like bodily sensations in panic disorder and worries in GAD.

Twelve-month prevalence rates for phobic anxiety disorders have been reported to range from 0.3 to 1.6% for Agoraphobia, 1.2 to 6.8% for Social Phobia, and 3.5 to 8.7% for Specific Phobia (Bijl et al., 1998; Alonso et al., 2004; Kessler et al., 2005; Stein et al., 2017; Wardenaar et al., 2017; Stagnaro et al., 2018). Lifetime prevalence rates have been reported to range from 0.9 to 3.4% for Agoraphobia, 2.4 to 7.8% for Social Phobia, and 7.7 to 10.1% for Specific Phobia (Bijl et al., 1998; Alonso et al., 2004; Kessler et al., 2005; Stein et al., 2017; Wardenaar et al., 2017). Prevalence rates for the subtypes of Specific Phobia have been reported to range from 3.3 to 5.7% for animal phobia, 4.9 to 11.6% for natural environment phobia (with 3.1 to 5.9% for height phobia), 5.2 to 8.4% for situational phobia (with 2.5 to 2.9% for flying phobia), and 3.2 to 4.5% for blood, injury and injection phobia (LeBeau et al., 2010).

With evidence from prospective studies, anxiety disorders must be seen as chronic disorders, starting in childhood, adolescence or early adulthood with a peak in middle age and a decrease in older age (Bandelow and Michaelis, 2015). According to the Global Burden of Disease Study 2015, anxiety disorders are ranked as the ninth largest contributor to global disability, leading to a global total of 24.6 million years lived with disability (YLD) in 2015 (Vos et al., 2016). For Specific Phobia, 18.7% of people with a 12-month Specific Phobia diagnosis reported severe role impairment in at least one out of four domains consisting of home, work, relationships and social life, and a mean number of 12.2 days out of role in the past year was assessed due to the disorder (Wardenaar et al., 2017). For Social Phobia, 37.6% of people with a 12-month diagnosis stated a severe role impairment in at least one domain, and a mean number of 24.7 days out of the role per 1 year was reported (Stein et al., 2017). For Panic Disorder with Agoraphobia, 84.7% of people with a 12-month diagnosis described severe role impairment, and for Agoraphobia without a history of Panic Disorder, but including panic attacks, 39.0% reported severe impairment (Kessler et al., 2006).

#### Exposure Therapy

The first-line treatment for anxiety disorders consists of exposure therapy (Chambless et al., 1998; Wolitzky-Taylor et al., 2008; Bandelow et al., 2014; Barlow et al., 2015; Steinman et al., 2015). During exposure therapy, patients confront themselves over a long period of time, repetitively, with a feared external or internal stimulus until distress has decreased significantly. They are also advised not to use cognitive or behavioral avoidance strategies. During exposure therapy in phobic anxiety disorders, patients particularly confront themselves with external stimuli such as height in fear of heights, crowds in Agoraphobia, or giving a speech in front of an audience in Social Phobia. This can be conducted in their imagination (exposure *in sensu*) or in real live (exposure *in vivo*). Exposure therapy in other anxiety disorders differs slightly from the procedure in phobic anxiety disorders. In panic disorder, interoceptive exposure to internal stimuli in the form of bodily sensations like heartbeat or dizziness are mainly applied (see for e.g., Forsyth et al., 2008; Gerlach and Neudeck, 2012). In Agoraphobia, interoceptive exposure is used in addition to *in vivo* exposure to external stimuli. In GAD treatment, patients confront themselves with internal or external aspects of their anxiety (Overholser and Nasser, 2000; Hoyer and Beesdo-Baum, 2012). Through imaginal exposure, GAD patients are exposed to central worries (e.g., concerning physical injury or impaired health), and through *in vivo* exposure, patients expose themselves to daily-live situations that elicit worries while not using safety behaviors such as telephone calls. In PTSD, a stress-related disorder, imaginal exposure to traumatic memories is performed and can be combined with *in vivo* exposure to daily-life actions (e.g., a patient traumatized by a car accident drives a car) (Riggs et al., 2007; Friedman, 2015). Besides anxiety disorders and PTSD, exposure therapy is also conducted in other disorders like obessive compulsive disorder (Lewin et al., 2014), eating disorders (see for e.g., Griffen et al., 2018; Waller and Raykos, 2019), or substance addiction (Marlatt, 1990; Drummond et al., 1995), respectively, with a modified procedure.

With phobic anxiety disorders as the focus of this systematic review and meta-analysis, there is robust empirical evidence for the efficacy of exposure therapy, even as the sole treatment method. According to numerous studies, *in vivo* exposure shows high effect sizes in the treatment of Agoraphobia (Ruhmland and Margraf, 2001), Social Phobia (Mayo-Wilson et al., 2014) and Specific Phobia (Wolitzky-Taylor et al., 2008). The most approved mechanisms underlying exposure treatment are habituation, extinction, correction of negative beliefs, and emotional processing (Foa and Kozak, 1986; Clark, 1999). Above that, inhibitory learning was recognized to be central to extinction learning (Craske et al., 2008). The authors propose that fear toleration, the development of competing non-threatening associations, and the enhancement of the accessibility and retrievability of those associations from different context and time, are more important for corrective learning than fear levels and fear reduction during exposure (Craske et al., 2008). Exposure is often performed in combination with further cognitive behavioral therapy (CBT) interventions such as psychoeducation, cognitive interventions, or relapse prevention strategies. While for Specific Phobia such additional interventions are minimal in many approaches, e.g., in the very effective One-Session Treatments (Davis et al., 2012), exposure in Social Phobia is typically integrated in further cognitive behavioral interventions and is particularly framed as experimental tasks, focusing on the verification and correction of dysfunctional beliefs in social situations (Clark, 2001).

Despite its convincing theoretical and empirical foundation, there seem to be barriers in the dissemination of exposure therapy in routine care. Neudeck and Einsle (2012) mention structural barriers (e.g., time, insurance, or logistics) and barriers up to the therapist (e.g., negative attitudes toward exposure therapy or insufficient familiarity with the method). Both impede the (accurate) application of exposure techniques in clinical practice. These barriers cause a problem for patients, preventing them from receiving highly efficacious treatment.

#### Virtual Reality Exposure Therapy

The use of Virtual Reality (VR) technology represents an option with the potential to overcome such barriers. VR exposure therapy (VRET), also called exposure therapy *in virtuo*, is based on the very similar rationale of *in vivo* exposure therapy, however, in VR exposure, phobic stimuli are presented to the patient in VR. VR is a computer-generated presentation, which provides input to the user's sensory system and interacts with the user (also see Diemer et al., 2015). Visual VR stimuli are presented via VR glasses (HMD: head mounted display) or via projection-based systems like CAVE-systems (cave automatic virtual environment), which is a room with up to six projection sides. Auditory input is applied via loudspeakers or earphones, and tactile, haptic or olfactory stimulation is possible but rarely provided. The aim is to replace sensory input from the real world and to create a presence of the user in the virtual world. To interact with the user in real time, the VR system collects information about the users' position and (head) movements via sensors and input devices like a head tracking system or a joystick.

By bringing virtual phobic stimuli into the therapist's office, VR exposure has many structural advantages. It is less time consuming in its application (e.g,. because driving to a high tower in heights phobia treatment is not necessary any more), cost-effective (e.g., in comparison to cost-intensive *in vivo* treatments for fear of flying), and requires less organization (e.g., regarding the acquisition of living spiders in spider phobia treatment, or of an audience for Social Phobia treatment). Furthermore, there are fewer difficulties concerning safety and insurance arrangements.

Above that, the VR technique might enhance usage of exposure treatment through a higher acceptance by patients, and thereby ease an efficacious procedure of psychotherapy. For *in vivo* exposure in Specific Phobia, high treatment responses but low treatment acceptance and high dropout rates have been reported in the past (Choy et al., 2007). In a direct comparison, García-Palacios et al. (2007) found evidence that patients with Specific Phobia prefer VR exposure to *in vivo* exposure and are significantly more willing to participate in VR treatment, mostly because they are too afraid of confronting the real feared stimuli. Quero et al. (2014) examined patients with Panic Disorder and Agoraphobia concerning their opinion toward VR and traditional interoceptive exposure before, directly after, and 3 months after treatment. Both treatments were well-accepted at all three time points, although VR exposure was considered a little, but not significantly, less aversive. Before treatment, the VR exposure rationale was expected to be significantly more logical and useful in other problems. Interestingly, higher expectations before treatment predicted a better clinical improvement at the post-test and follow-up. After 3 months, participants in the traditional interoceptive exposure group reported a significantly higher satisfaction. Nevertheless, clinical improvements did not show significant differences between the two conditions at the post-test and follow-up. Concerning dropout from an ongoing exposure treatment, a meta-analysis of randomized-controlled trials (RCTs) conducted by Benbow and Anderson (2018) found no significant difference in the likelihood of discontinuation between VR and *in vivo* exposure, although the attrition rate for VR exposure was found to be slightly below estimates reported for *in vivo* exposure and CBT for anxiety disorders. Thus, offering VR exposure might, in particular, lead to a higher number of patients agreeing to exposure therapy. During and after exposure therapy, on the other hand, an application in VR might not have relevant advantages with regards to dropout rates—at least if patients were randomly assigned to either VR or *in vivo* therapy—or with regards to the patients' opinion toward the treatment.

Besides the patients' acceptance, VR provides the advantage that phobic objects and situations can be easily adapted according to therapeutic considerations. For example, the therapist can entirely control type, intensity, duration and repetition of the exposed object or situation, and can implement specific stimuli (e.g., turbulences in the exposure of flight phobia) (Diemer et al., 2015). Furthermore, contextual shifts are less time consuming and costly in VR in comparison to *in vivo* exposure (Botella et al., 2017), what might be relevant as using multiple contexts in spider phobia already showed an improvement in the generalizability of exposure therapy (Shiban et al., 2015). Altogether, VR provides a high level of control and flexibility with the possibilities to even surpass reality (Botella et al., 2017). One example is the use of virtual spiders, which can be constructed to be much bigger than living spiders (see for e.g., Shiban et al., 2015). These possibilities might even facilitate an enhancement of the efficacy of VR in comparison to *in vivo* exposure therapy, although empirical evidence from studies explicitly exhausting the technical possibilities of VR exposure are still rare.

Finally, the German Practice Guideline for anxiety disorders already recommends VR exposure on the basis of expert consensus for Specific Phobia if *in vivo* exposure is not available or possible (Bandelow et al., 2014). Moreover, the guideline preliminarily lists VR therapy as an effective treatment option for Agoraphobia/Panic Disorder.

#### Efficacy of Virtual Reality Exposure Therapy for Phobic Anxiety Disorders

To empirically prove the efficacy of VR exposure therapy in anxiety disorders, numerous original studies and meta-analyses have been published throughout the last decade. While some of the meta-analyses highlight a broad perspective on the use of VR in cognitive behavioral therapy, including other VR-based interventions than exposure (Fodor et al., 2018), or show effect sizes for symptom improvements through VR exposure under the inclusion of primary studies that applied no control group (Parson and Rizzo, 2008), most meta-analyses focus on comparisons of VR exposure to inactive and active control conditions. According to the Cochrane Handbook for Systematic Reviews of Interventions, inactive control groups consist of for example a placebo, no treatment, standard care, or a waitlist control, while active control groups consist of a different kind of therapy (Higgins and Green, 2011). Results from previous meta-analyses on the efficacy of VR exposure therapy for anxiety disorders showed that VR exposure therapy yields large effects with regards to the reduction of anxiety symptoms (Parson and Rizzo, 2008) and greatly outperforms inactive control conditions (Powers and Emmelkamp, 2008; McCann et al., 2014; Fodor et al., 2018; Carl et al., 2019). Compared to active treatment conditions, results were more indifferent. Two meta-analyses showed no significant difference in the efficacy of VR exposure for anxiety disorders in comparison to classical evidence-based treatments like CBT, imaginal exposure and *in vivo* exposure (Opriş et al., 2012), or when specifically compared to *in vivo* exposure therapy (Carl et al., 2019). In contrast, Fodor et al. (2018) found that non-VR interventions like CBT, imaginal exposure, and *in vivo* exposure were slightly more effective than VR exposure in anxiety disorders. Powers and Emmelkamp (2008) instead reported a small effect size favoring VR exposure over *in vivo* exposure for anxiety disorders. As further results, Opriş et al. (2012) showed that gains from VR exposure therapy could be transferred to real life situations, and that VR exposure showed a good stability of its outcomes over time, similar to that of classical evidence-based treatments, yet, there is evidence that deterioration rates of VR therapy for anxiety disorders did not differ significantly from other therapeutic approaches and were less frequent in comparison to waitlist control groups (Fernández-Álvarez et al., 2018).

In addition to addressing different anxiety disorders, some meta-analyses focused on the efficacy of VR exposure in specific kinds of Phobias. Morina et al. (2015) conducted a meta-analysis on fear of heights and fear of spiders in particular. The examination of behavior changes in real life situations and stability over time showed that VR exposure performed significantly better than waitlist did as an inactive control condition, and that there were no significant differences between VR exposure therapy and *in vivo* exposure therapy as an active control condition. Cardoş et al. (2017) conducted a meta-analysis on flight phobia in particular. They reported an advantage of VR exposure therapy when compared to classical evidence-based-interventions at the post-test and follow up, and when compared particularly to imaginal or *in vivo* exposure at follow up but not at post-test. In a meta-analysis on Social Phobia in particular, Chesham et al. (2018) showed no relevant difference between VR and imaginal or *in vivo* exposure.

Notably, there were different primary studies included in the reported meta-analyses. Regarding the meta-analyses addressing different anxiety disorders, Parson and Rizzo (2008) examined the effects of VR exposure therapy for Phobias and PTSD in studies without a control group, with waitlist, bibliotherapy, relaxation, or attention as inactive control groups, or with *in vivo* exposure as an active control group. The meta-analysis by Fodor et al. (2018) provides a broader perspective on the use of VR in cognitive behavioral therapy and in this regard examined RCTs on VR-enhanced exposure and also on VR-enhanced CBT interventions without exposure. However, in two particular subgroup analyses, VR-enhanced exposure only was compared to inactive control conditions including waitlist, placebo, treatment-as-usual, and relaxation, and to active control conditions including CBT, imaginal exposure, and *in vivo* exposure. Carl et al. (2019) synthesized trials on anxiety disorders and PTSD with random or matched allocation and compared VR exposure conditions, in which VR was not combined with another intervention, medication, or placebo to mixed control conditions like wailtlist, information, attention control, treatment as usual, relaxation, or present-centered therapy, and to *in vivo* exposure as an active control condition. McCann et al. (2014) synthesized RCTs on different anxiety disorders and compared VR exposure to waitlist or active placebo as inactive control groups, and to active control groups which in this study constisted of interventions like treatment as usual, cognitive therapy, present centerd therapy, computer-aided exposure, CBT, imaginal exposure, or *in vivo* exposure. Powers and Emmelkamp (2008) examined RCTs on anxiety disorders and PTSD and compared VR conditions that do not combine VR with other interventions or medication, to inactive control groups like waitlist, attention control, bibliotherapy, or relaxation, and to *in vivo* exposure as active control group. Opriş et al. (2012) examined RCTs on anxiety disorders comparing VR conditions to waitlist as inactive control group, and to classical evidence-based treatments like CBT, imaginal exposure and *in vivo* exposure as active control groups in a clinical population.

Regarding the reported meta-analyses addressing particular Phobias, Morina et al. (2015) synthesized studies on Specific Phobia and compared the efficacy on behavioral outcome measures in VR based exposure interventions with inactive control conditions like waitlist or attention placebo, and with active control conditions like CBT, imaginal exposure or *in vivo* exposure. Cardoş et al. (2017) included RCTs on flight phobia and compared VR exposure treatments with or without other interventions, to waitlist or attention control as inactive control groups, and to classical evidence-based interventions like CBT, bibliotherapy, cognitive therapy, relaxation, CBT with standard exposure (*in vivo*), relaxation techniques with imaginal exposure, and computer aided exposure as active controls, and particularly to exposure-based interventions including imaginal and *in vivo* exposure as active control groups. Chesham et al. (2018) included studies on Social Phobia with random, quasi-random or matched assignment, and compared VR exposure conditions to waitlist as inactive control group, and to *in vivo* or imaginal exposure as active control conditions.

Overall, only two studies (Powers and Emmelkamp, 2008; Carl et al., 2019) conducted a quantitative meta-analysis on the efficacy of VR exposure therapy in comparison to *in vivo* exposure therapy as the gold standard treatment for phobic anxiety disorders. No previous meta-analysis considered the amount of exposure applied in VR and *in vivo* conditions. This reduces the internal validity of previous results, because differences in effect sizes between VR and *in vivo* exposure therapy cannot be clearly attributed to the application mode of exposure treatment but could be due to differences in the load of exposure treatment.

### Objectives

As the first systematic review and meta-analysis, we aim at comparing the efficacy of VR and *in vivo* exposure therapy for phobic anxiety disorders, based on randomized controlled trials including an equivalent amount of exposure in VR and *in vivo*. We chose to focus on phobias, as they are a highly comparable group of anxiety disorders with a similar procedure of exposure treatment. In these disorders, *in vivo* exposure as an individual treatment component is considered the gold standard. Furthermore, there are concrete external phobic stimuli that are usually presented during *in vivo* exposure, and these stimuli can be directly transferred to VR.

In our quantitative meta-analysis, we evaluate pre to post effect sizes for VR exposure therapy, *in vivo* exposure therapy, and for the comparison of VR and *in vivo* exposure therapy. Furthermore, we report the individual effect sizes of all included studies and provide a systematic review of the participants' characteristics, the materials, and the treatment procedures used. On this basis, we aim at discussing potential mechanisms of more or less efficacious VR exposure therapy.

Other than the recent meta-analysis by Carl et al. (2019), which provides a broad overview of the topic, our focus is the direct comparison of an equivalent amount of exposure in VR and *in vivo*. In this regard, we apply stricter inclusion criteria than Carl et al. (2019) to control for potential confounding variables. We exclude not only studies with a different amount of exposure in the VR and *in vivo* condition, but also studies with imaginal exposure but no *in vivo* exposure as the control group, with exposure treatment applied only to selected participants, with VR presentation without using immersive systems (e.g., HMD) and head tracking, and with dependent samples. Since Carl et al. (2019) do not provide a qualitative review of their included studies, we furthermore fill this gap. We therefore offer detailed descriptions and assessments of the individual studies' characteristics and of differences in their individual effect sizes. We summarize the patients' characteristics as well as the treatment materials and procedures, including information on the exposure strategy, the type of HMDs and their technical features, the virtual and *in vivo* environments, and additional interventions along with the exposure that were applied in the VR and *in vivo* exposure condition. As VR exposure is a quickly expanding field, high quality meta-analyses and high resolutions in research is needed to contribute to theory building, the development of future research questions, and the improvement of VR exposure procedures.

### Research Question

We examine whether there is a relevant difference in the efficacy of VR exposure therapy in comparison to *in vivo* exposure therapy as the gold standard treatment for phobic anxiety disorders, when synthesizing RCTs with an equivalent amount of exposure in the VR and *in vivo* condition. Furthermore, we aim at a qualitative examination of the participants' characteristics, materials, and treatment procedures of all the included studies.

## Methods

### Protocol

We used the Preferred Reporting Items for Systematic Reviews and Meta-Analyses (PRISMA) checklist and protocol provided by the PRISMA Group (Moher et al., 2009).

### Eligibility Criteria

Only original studies published until June 2019 were included. The language inclusion criterion was (1) a report written in English or German. The population inclusion criterion was (2) an ICD or DSM diagnosis for Agoraphobia, Specific Phobia, or Social Phobia. The intervention inclusion criteria were (3) a treatment for Agoraphobia, Specific Phobia, or Social Phobia, (4) exposure therapy in virtual reality using immersive systems (e.g., HMD) and head tracking (no augmented reality or 3D computer animation in front of a PC screen) in the experimental group, (5) exposure therapy *in vivo* in the control group, and (6) no combination of the VR or *in vivo* exposure therapy with a specific psychopharmacological treatment. The outcomes inclusion criterion was that (7) studies examined the reduction of phobic anxiety as the primary outcome. To ensure an high internal and external validity, the inclusion criteria for study design were (8) a minimum number of 10 participants per group, (9) a randomized assignment of the participants to one of both exposure conditions, (10) an equivalent amount of exposure in both conditions and equivalency concerning additionally applied interventions alongside exposure treatment, (11) a pre and post measurement of phobic anxiety (12) with a symptom specific, standardized questionnaire or interview, and (13) sufficient statistical values (means and standard deviations in outcome parameters for each group).

### Information Sources

A literature search in PubMed, PsychInfo and Web of Science was conducted in October and November 2017 and was updated in November 2018 and in June 2019. We also asked experts in the field of VR therapy to provide possible eligible studies.

### Search

We searched for the keywords “virtual” and “phobia” in the PubMed, PsychInfo and Web of Science databases. Moreover, we conducted a search on the term “social anxiety.” We did not set a time limit for the period in which the studies were conducted. Depending on the different databases' search template structure, we used slightly different search strategies. In PubMed the connector ‘AND’ was used to search for “Virtual AND Phobia” as well as “Virtual AND anxiety AND social” in titles and abstracts. In PsycInfo we searched for “virtual” in title and “phobia” in abstracts, as well as for “virtual” in title and “anxiety” and “social” in abstracts. In Web of Science we searched for “virtual” in title and “phobia” in the topic, as well as for “virtual” in title and “anxiety” and “social” in the topic.

### Study Selection

**Figure 1:**
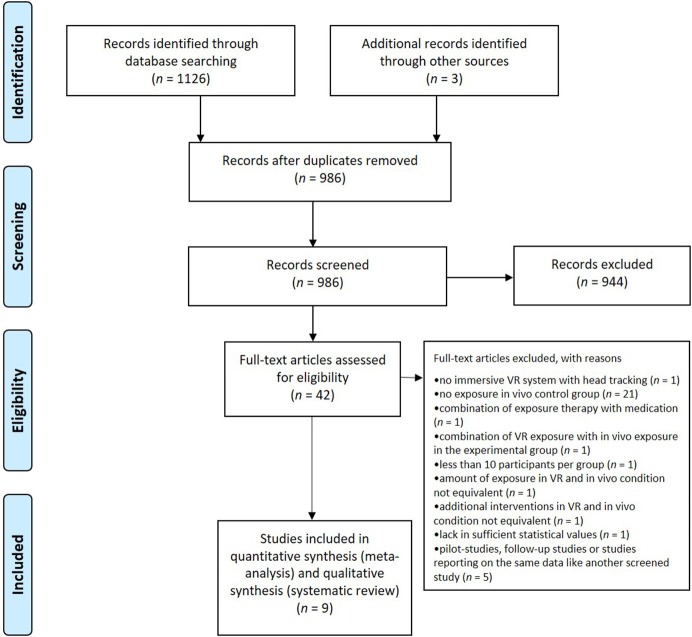
PRISMA flow diagram (Moher et al., 2009) reporting the number of screened studies and the number of studies excluded during the screening process.

A PRISMA flow diagram (Moher et al., 2009) illustrates the number of studies screened and excluded during the screening process (see Figure 1). Therefore, the numbers from the first search in November 2017 and the updated search in November 2018 and June 2019 were summed up. During screening process of all records identified through database searching (*n* = 1,126) and other source (*n* = 3), obvious duplicates (*n* = 143) were removed first. The titles and abstracts of the remaining reports (*n* = 986) were then screened against the eligibility criteria. If a title or abstract provided the information that at least one eligibility criterion was not fulfilled, the record was excluded (*n* = 944). All remaining records with no evidence for a violation in eligibility criteria within the abstract were passed on for full-text screening (*n* = 42). During this process, all 13 eligibility criteria were assessed, and reports not fitting every eligibility criterion were excluded (*n* = 33). We contacted two authors to check on dependent samples in different records to avoid including data twice. One author provided the information that there was no overlap in the samples of two flight phobia studies (Rothbaum et al., 2000, 2006). The other author informed us that one eligible record (Robillard et al., 2010) includes preliminary data from a larger study (Bouchard et al., 2016), so we excluded the preliminary data from our meta-analysis.

There were three researchers involved in the screening process. One researcher screened the titles and abstracts of all studies, and then screened the full-text of the remaining studies providing suggestions for the selection of eligible reports. A second researcher additionally screened the abstracts and full-text and selected eligible reports. Disagreements concerning the inclusion of studies after the full-text screening of two researchers were discussed with the third researcher. We performed the exclusion process based on the information provided in the published articles and to the best of our knowledge.

### Data Collection Process

Data were extracted from each report independently by two researchers. Means and standard deviations for the participants' age and the distribution of sexes was missing in one report, but we could not reach the respective author. One author was contacted concerning missing information on the type of HMD and the author provided the respective information. Technical data on the image resolution and the field of view of HMDs were collected from the reports, and if not available HMD data sheets from internal databases and from the producers' websites were used. Because not all reports provided statistical data on both, the intent-to-treat and the completer sample, we contacted the respective authors to ask for additional data. As we could not receive both data sets for every included study, we used the intent-to-treat data if available, otherwise we used data from the completer sample. Disagreements between the two researchers concerning the collected data were discussed with the third author until a consensus was reached.

### Data Items

The following data were extracted: (1) number of participants in total and in the VR and *in vivo* group, (2) age of the participants as means, standard deviations and range, (3) distribution between the sexes of the participants, (4) medication of the participants, (5) treated disorder (Agoraphobia, Social Phobia, or Specific Phobia), (6) number of total treatment sessions in the VR and *in vivo* condition (exposure sessions plus additional sessions applying other interventions), (7) amount of exposure in the VR and *in vivo* condition, assessed in form of (7a) the number of exposure sessions and (7b) the duration of exposure sessions in minutes, (8) exposure strategy, (9) type of HMD with (9a) resolution and (9b) field of view, (10) information on movement mode and further stimulation of senses alongside the sense of sight in VR, (11) description of the VR exposure environment, (12) description of the *in vivo* exposure environment, (13) therapeutic interventions used for pre- and post-processing and to accompany exposure, (14) sample on which the calculation of means and standard deviations was carried out on (intent-to-treat vs. completer sample), (15) type of standardized measurement for symptom specific anxiety, and (16) means and standard deviations of the pre- and post-symptom measurement for the VR and *in vivo* group. If more than one symptom measurement was applied, the measurement that assessed the anxiety symptoms of the treated phobia the most specifically was collected.

### Risk of Bias in Individual Studies

Risk of bias in the individual studies was assessed using a tool for bias detection in randomized trials from the Cochrane Collaboration (Higgins et al., 2011). To assess the risk of selection bias, performance bias, detection bias, attrition bias, and reporting bias, we checked the criteria (1) random sequence generation, (2) allocation concealment, (3) blinding of participants and researchers, (4) blinding of outcome assessment, (5) incomplete outcome data, and (6) selective reporting. The risk of bias in each domain was rated as either low, unclear, or high following the explanations and examples provided by Higgins et al. (2011). We again performed this process based on the information provided in the published articles and to the best of our knowledge.

### Synthesis of Results

#### Qualitative Review

As qualitative synthesis of all included studies, we conducted a qualitative review. A qualitative review provides a structured presentation and assessment of central characteristics of the included studies. For this purpose, we examined and summarized the participants' characteristics, diagnostic measures, study methodology, and treatment materials and procedures to provide an overview and a basis for discussions on effect sizes and future research perspectives. In the examination of the treatment materials and procedures, we particularly considered information on the exposure strategy, visual VR devices (type of HMDs including technical data on resolution and field of view), movement mode in VR, devices for further stimulation of senses alongside the sense of sight in VR, VR and *in vivo* exposure environments, and additional interventions alongside the exposure applied in the VR and *in vivo* condition (see collected data items in section Data Items).

#### Quantitative Meta-Analysis

To provide a statistical summary of the results on the efficacy of VR and *in vivo* exposure therapy from the included studies on phobic anxiety disorders, we performed a quantitative meta-analysis. In this regard, we calculated pre- to post-effect sizes for VR exposure, *in vivo* exposure, and the comparison of VR to *in vivo* exposure for the individual studies and then synthesized them for all included studies. In addition, we separately calculated synthesized effect sizes of all studies on Specific Phobia, Social Phobia and Agoraphobia. We used Microsoft Word Excel 2016 as the software tool for the statistical analysis.

##### Effect sizes for the individual studies

*VR exposure therapy and in vivo exposure therapy*. As a first step, we calculated the pre- to post-effect sizes for (1) the VR exposure treatment and (2) the *in vivo* exposure treatment of the individual studies included in the meta-analysis. We therefore computed the standardized mean difference between pre- and post-measurement separately for the VR group and *in vivo* group of each study using Cohen's *d* for studies that use pre-post-scores according to Borenstein et al. (2009). Because correlations between the outcome measures were not available, the value was set to zero, constituting a conservative calculation (Lenhard and Lenhard, 2014). As an indicator corrected for small sample bias, we computed the Hedges' *g* (Hedges and Olkin, 1985). The Hedges' *g* coefficients were calculated by the multiplication of Cohen's *d* and a correction factor according to Borenstein et al. (2009). In addition, variance, standard error and 95% confidence interval for Hedges' *g* were calculated. The Hedges' *g* may be interpreted as small (0.2), medium (0.5), and large (0.8) (Ellis, 2010).

*Comparison of VR and in vivo exposure therapy*. As a second step, we calculated the pre- to post-effect sizes for (3) the comparison of VR to *in vivo* exposure therapy for the individual studies included in the meta-analysis. For each study, we calculated the standardized mean difference by subtracting the pre to post change in the *in vivo* group from the pre to post change in the VR group, and then divided the result by the pooled pre-test standard deviation (Morris, 2008). The standard deviations were pooled across pretest scores of both conditions as recommended by Morris (2008) as the best choice for pretest-posttest-control group designs. Hedges' *g* again was calculated for the standardized mean difference using a correction factor (Morris, 2008; Borenstein et al., 2009). The variance, standard error and confidence interval for *g* were also calculated. The variance of *g* was computed as the multiplication of the squared correction factor and an approximation of the variance of the uncorrected standardized mean difference, using an equation for independent samples following Borenstein et al. (2009). In this calculation, a positive Hedges' *g* effect size reflects superiority of the VR exposure treatment, while negative coefficients indicate superiority of the *in vivo* exposure treatment.

##### Synthesis of effect sizes for all studies on phobic anxiety disorders

To synthesize the effect sizes for VR exposure, *in vivo* exposure, and the comparison of VR to *in vivo* exposure therapy from all included studies on phobic anxiety disorders, we estimated total mean effect sizes in a random-effect model following Borenstein et al. (2009). A random-effect model accounts for the variation across the studies and assumes that the true effects are normally distributed (Borenstein et al., 2009). It therefore considers the within-study variance and the variance between-studies.

Three random-effect models were calculated to synthesize the individual effect sizes for (1) VR exposure, (2) *in vivo* exposure, and (3) the comparison of VR and *in vivo* exposure from all included studies. During the calculation of each model according to Borenstein et al. (2009), an estimate for the between-studies variance was computed first, using the method of moments. Second, each study was weighted by the inverse of its variance plus the estimated between-studies variance. Third, we estimated the mean effect size. For this purpose, we calculated the weighted mean of the Hedges' *g* effect sizes of all studies, as the sum of the weighted effect sizes of the individual studies, divided by the sum of the weights. We also computed the variance, standard error, confidence interval, *Z*-value and two-tailed *p*-value for the estimated mean effect size.

##### Synthesis of effect sizes for studies on Specific Phobia, Social Phobia, and Agoraphobia

In addition, we calculated synthesized effect sizes for VR exposure, *in vivo* exposure, and for the comparison of VR to *in vivo* exposure therapy separately for studies on Specific Phobia, Social Phobia, and Agoraphobia. Because the estimate of the between-studies variance, which is necessary to calculate the random-effect model, has a poor precision if the number of studies is very small (Borenstein et al., 2009), we calculated a fixed-effect model as an option for a small number of studies suggested by Borenstein et al. (2009). A fixed-effect model is already reasonable for a synthesis up from two studies, because a synthesis of two or more studies offers a more precise estimate of the true effect compared to one study alone (Borenstein et al., 2009). A fixed-effect model does not allow inferences on a wider population but provides a descriptive analysis about the included studies (Borenstein et al., 2009). It assumes that the true effect size is the same in all studies included in the meta-analysis (Borenstein et al., 2009). Although the fixed-effect model actually demands functionally identical studies (Borenstein et al., 2009), which is basically implausible in studies performed by different researchers, it does however seem applicable for the synthesis of studies on one kind of phobic anxiety disorder in this meta-analysis, particularly because the inclusion criteria created a relatively high homogeneity concerning the participants and the procedure used across the studies.

The fixed-effect models were computed according to Borenstein et al. (2009). Altogether, we calculated nine fixed-effect models synthesizing the pre to post effect sizes for (1) VR exposure therapy, (2) *in vivo* exposure therapy, and (3) the comparison of VR to *in vivo* exposure therapy separately, for all included studies on (1) Specific Phobia, (2) Social Phobia, and (3) Agoraphobia. During the calculation of each fixed-effect model, the effect size of each individual study was weighted by the inverse of its own variance. The weighted mean was then calculated as the sum of the weighted effect sizes, divided by the sum of the weights. In addition, we computed the variance, standard error, 95% confidence interval, *Z*-value and two-tailed *p*-value for the summary effect using equations according to Borenstein et al. (2009).

### Risk of Bias Across Studies

To assess the risk of bias across the included studies, a funnel plot with the standard errors for Hedges' *g* on the axis of ordinates and Hedges' *g* on the axis of abscissae was conducted. We therefore used Hedges' *g* for the comparison of the VR and *in vivo* condition, as the main result of our analysis. A skewed or asymmetrical funnel in a visual examination can indicate a publication bias, as (smaller) studies that do not show statistically significant effects remain unpublished (Easterbrook et al., 1991; Egger et al., 1997).

## Results

### Study Selection

The PRISMA flow-chart diagram (Figure 1) shows the number of screened studies, excluded studies, and studies finally included in the meta-analysis. During full-text assessment, 33 studies were excluded because they did not fulfill the eligibility criteria for the following reasons: presentation of 3-D-stimuli on a PC screen instead of VR presentation using immersive systems (e.g., HMD) and head tracking (Klinger et al., 2005), comparison of two different VR exposure groups but no comparison to an *in vivo* exposure control group (Fraser et al., 2001), imaginal/*in sensu* exposure but no *in vivo* exposure as the control group (Wiederhold et al., 2001, 2002; Wallach et al., 2009; Rus-Calafell et al., 2013; Triscari et al., 2015), interoceptive exposure but no *in vivo* exposure as the control group (Quero et al., 2014), interoceptive and imaginal exposure but no *in vivo* exposure as the control group (Vincelli et al., 2003), computer-aided exposure as the control group (Tortella-Feliu et al., 2011), *in vivo* exposure only for patients with comorbid conditions in the control group (Krijn et al., 2007), relaxation training as the control group (Mühlberger et al., 2001), cognitive treatment as the control group (Mühlberger et al., 2003; Wallach et al., 2011), evaluation of VR exposure treatment effects on a graduation flight conducted accompanied or alone but no comparison between VR exposure and *in vivo* exposure as control group (Mühlberger et al., 2006), no control group (Baños et al., 2002; Anderson et al., 2003, 2005; Wald, 2004; Grillon et al., 2006; Piercey et al., 2012; Felnhofer et al., 2014), combination of exposure with paroxetine (Pitti et al., 2015), <10 participants per group (Botella et al., 2000), no equivalent amount of exposure in the VR and *in vivo* condition (Pelissolo et al., 2012), report of a study protocol without results (Miloff et al., 2016), and no equivalency concerning additional interventions applied alongside exposure in the VR and *in vivo* condition (Miloff et al., 2019). In the latter reports by Miloff et al. (2016, 2019), VR exposure was conceptualized as a fully automated VR serious game constructed to work independently from the presence of a human therapist. In contrast, *in vivo* exposure was conducted as a single session exposure approach according to Öst and was guided by a human therapist. Therefore, the VR condition was confounded with an automated exposure approach. As the *in vivo* exposure condition furthermore consisted of additional interventions conducted by the human therapist, that were not applied in the VR condition, like reflection on catastrophic beliefs, exploration of what occurs at each treatment stage, exploration of violations of expectancy and monitoring and discussion of safety behaviors, the study did not fulfill our inclusion criteria concerning equivalence in the additional interventions applied alongside exposure in the VR and *in vivo* condition and was thus excluded from the analysis. The following studies were also excluded for the exceptional reasons of a combination of VR exposure with *in vivo* exposure (Choi et al., 2005), and as pilot trials, follow-up studies, or studies that examined a new research question based on the data of another screened study (Rothbaum et al., 2002; Robillard et al., 2010; Safir et al., 2012; Anderson et al., 2016; Kampmann et al., 2019).

### Study Characteristics

Nine studies fulfilled all eligibility criteria and were included in our meta-analysis (see Tables 1, 2 for study characteristics). The final sample consisted of two studies on Agoraphobia (Botella et al., 2007; Meyerbroeker et al., 2013), three on Social Phobia (Anderson et al., 2013; Bouchard et al., 2016; Kampmann et al., 2016), and four on Specific Phobia (Rothbaum et al., 2000, 2006; Emmelkamp et al., 2002; Michaliszyn et al., 2010). As different sub-types of Specific Phobia, two studies target fear of flying (Rothbaum et al., 2000, 2006), one study targets fear of heights (Emmelkamp et al., 2002), and one study targets fear of spiders (Michaliszyn et al., 2010).

**Table 1:** Participants' and treatment characteristics in RCTs included in the meta-analysis.

	**Participants**						**Treatment**		
**References**			**Age in years**		**Sex (m/f)**	**Medicated**	**Total sessions**	**Exposure sessions (VR and** ***in vivo*** **condition)**	
	***N***	**ICD or DSM diagnosis**	***M* (*SD*)**	**range**	***n* or %**	***n* or %**	***N***	***n***	**min./session**
Rothbaum et al., 2000	30	Specific Phobia (fear of flying)^a^	40.55 (10.64)^e^	24–69	29%/71%^e^	N/A	8	4	N/A
Emmelkamp et al., 2002	33	Specific Phobia (fear of heights)	43.97 (9.34)	N/A	18/15	N/A	4	3	60
Rothbaum et al., 2006	58	Specific Phobia (fear of flying)^b^	VRET: 38.62 (9.16) IVET: 44.45 (12.16)	N/A	12/46	N/A	8	4	N/A
Michaliszyn et al., 2010	32	Specific Phobia (spider phobia)^c^	29.1 (7.99)	18–51	1/31	0	8	6	90
Anderson et al., 2013	69	Social Phobia	39.03 (11.26)^f^	19–69	38.1%/61.9%^f^	N/A^i^	8	VRET: 4 IVET: 6^k^	VRET: 30 IVET: 20
Kampmann et al., 2016	40	Social Phobia	VRET: 39.65 (11.77) IVET: 37.5 (11.27)^g^	18–65^g^	VRET: 35%/65% IVET: 25%/75%^g^	N/A	10	7	60
Bouchard et al., 2016	39	Social Phobia	VRET: 36.2 (14.9) IVET: 36.7 (11.1)	N/A	7/32	VRET: 2 IVET: 3	14	8	20–30
Botella et al., 2007	24	Agoraphobia^d^	34.7 (12.31)^h^	18–72	29.7%/70.3%^h^	66.6%^h^	9	6	60
Meyerbroeker et al., 2013	46	Agoraphobia	N/A	18–65	N/A	N/A^j^	10	6	60

*The left side of this table presents the number of patients included in the individual studies, their ICD or DSM diagnosis, their age, the distribution of the sexes, and the number of medicated patients. Age is reported as means and standard deviations either for the whole sample or separately for the both treatment groups VR exposure therapy (VRET) and in vivo exposure therapy (IVET). The range of age is stated for the whole sample. The distribution of sexes is presented as absolute numbers or as percentages of male and female participants and is reported either for the whole sample or separately for both treatment conditions. If information on medication was available, the absolute number or the percentage of medicated participants was reported either for the whole sample or separately for both treatment conditions. The right side of the table gives an overview of the treatment sessions applied to participants in the VR exposure and in the in vivo exposure condition. The total number of treatment sessions adds up the number of exposure sessions and the number of additional sessions for pre-and post-processing and to accompany exposure. The number of exposure sessions and the duration of one single exposure session in minutes is reported, too. A description of the concrete exposure procedures and interventions performed during additional sessions is summarized in Table 2. Studies are sorted by the type of phobia and date of publication. N (total participants) = 371. N/A: information was not available*.

a *Patients diagnosed with Agoraphobia with flying as main feared stimulus, n = 3*.

b *Patients diagnosed with Agoraphobia (with or without Panic Disorder) with flying as main feared stimulus, n = 10*.

c *Patients with partial diagnosis of Specific Phobia but scoring within the phobic range on questionnaire measures and BAT, n = 4*.

d *Participants with diagnosis of Panic Disorder without Agoraphobia in whole sample including waitlist, % = 17.1*.

e *Values for the whole sample including third condition (waitlist), N = 45*.

f *Percentages for whole sample including waitlist condition, N = 97*.

g *Participants included in VR and in vivo group by re-randomization from waitlist are not included in values for mean age and sex distribution, but in age range*.

h *Values for whole sample including third condition (waitlist)*.

i *Inclusion criteria consist of a stable medication for 3 months*.

j *Tranquilizers excluded, stable dose of antidepressants required*.

k *Different number and duration of exposure sessions but with the same total duration of 120 min in the VR exposure condition (four times 30 min) and in the in vivo exposure condition (six times 20 min)*.

**Table 2:** Treatment materials and procedures in the VR and *in vivo* exposure conditions.

		**Exposure Treatment**				
**References**	**Exposure strategy (VR and *in vivo* condition)**	**Type of HMD with image resolution and field of view**	**Movement mode in VR and devices for tactile and haptic stimulation**	**VR environments**	***In vivo* environments**	**Additional interventions (VR and *in vivo* condition)**
Rothbaum et al., 2000	Gradual; encouraging comments by therapist	VR6: 640 × 480/60°	Thunderseat^a^	Window seat inside the passenger compartment of a commercial airplane with empty seats; takeoffs and landings; flying in calm and stormy weather	Airport: ticketing, trains, parked planes, waiting area; sitting on stationary plane (+ imaginal exposure of take-offs, cruising, landing, etc. on stationary plane^c^)	Treatment planning and explaining the rationale to the patients, anxiety management techniques (breathing retraining, cognitive restructuring, thought stopping, in case of panic attacks: hyperventilation exposure)
Emmelkamp et al., 2002	Gradual; habituation rationale; verbal guidance and encouragement by therapist	Cybermind Visette Pro: 640 × 480/71.5°	Walk around freely on 1 m^2^; railing to hold on	Mall with four floors with escalators and balustrades, fire escape (height: ~50 feet), roof garden at top of building (height: ~65 feet)	Real locations corresponding to VR environments	Intake session
Rothbaum et al., 2006	Gradual	VFX3D: 640 × 480/35°	Seat with seatbelt and bass speaker underneath	Window seat inside the passenger compartment of a commercial airplane; start of engines, announcements of pilot and attendants, taking the plane to the runway, take-off, flying in bad and good weather, landing	Airport: ticketing, trains, waiting area; coordination center tower: viewing planes, speaking with knowledgeable airport personnel; sitting on stationary plane (+ imaginal exposure of take-offs, cruising, landing, etc. on stationary plane^c^)	Treatment planning, anxiety management techniques (breathing retraining, cognitive restructuring, thought stopping, interoceptive exposure)
Michaliszyn et al., 2010	Gradual	I-glasses PC/SVGA A502085® (i-O display systems): 800 × 600/26°	Handheld wireless gyration mouse	Three levels of animated spiders of different shapes and sizes; top item: large black-widow spider	Two types of spiders; top-item: manipulate them in the hand	Psychoeducation, cognitive restructuring, relapse prevention
Anderson et al., 2013	Gradual; habituation rational	VFX headset: 640 × 480/35°	N/A	Virtual conference room (about five audience members), virtual classroom (35 audience members), virtual auditorium (100 audience members); different audience reactions (interested, bored, supportive, hostile, distracted, etc.); audience members posing standardized or individualized questions	Group therapy with up to five participants, videotaped speech in front of the other group members, individualized positive feedback from other group members	Psychoeducation, realistic goal setting for social situations through techniques like cognitive preparation, challenging of cost and probability biases, relapse prevention, homework (daily mirror task, daily record of social situations, identification of cognitive bias)
Kampmann et al., 2016	Gradual; until anxiety decreased; communication with therapist in next room via intercom	nVisor SX: 1,280 × 1,024/60°	N/A	Giving a talk in front of an audience followed by questions, talking to a stranger, buying and returning clothes, attending a job interview, being interviewed by journalists, dining in a restaurant with a friend, having a blind date; semi-structured dialogues with different dialogue-styles and content (friendly vs. unfriendly; personal relevance), different number, gender and gestures of avatars	Participants' individual social situations which were translated to exposure exercises (e.g., in supermarkets, subway stations, cafés, etc.); or exposure in personal environment of the participants with contact to therapist via the telephone before and after the exposure	Therapy rationale and anxiety hierarchy, relapse prevention, evaluation of the therapy
Bouchard et al., 2016	Focus of the exposure: develop new, nonthreatening and adaptive interpretations; habituation not required; active modeling from the therapist in early sessions	eMagin z800: 800 × 600/40°	Wireless computer mouse	Speaking in front of audience in a meeting room, having a job interview, introducing oneself and having a talk with supposed relatives in an apartment, acting under the scrutiny of strangers on a coffee shop patio, facing criticism or insistence (meeting unfriendly neighbors, refusing to buy goods from a persistent seller at a store); preformatted answers triggered by the therapists	Role-playing and guided exposure inside or outside the therapist's office (e. g. asking for the time in a coffee shop, asking strangers on a date, giving an awkward impromptu speech to an audience of staff members, making improper requests in boutiques and stores); audience constituted by laboratory members	Developing a personal case conceptualization model, symptoms and avoidance/safety behavior, cognitive restructuring, relapse prevention
Botella et al., 2007	Gradual	V6: 640 × 480/60°	Mouse	Training room, house, subway, bus, shopping mall, tunnel; simulation of bodily sensations (palpitations and breathing difficulties with three levels of intensity from mild to accelerated, visual effects like tunnel vision, blurred vision, double vision); different modulations: number of people present, length of the trips, difficulties like problem with the credit card at the shopping mall or the elevator suddenly stopped between two floors etc.	*in vivo* exposure	Psychoeducation, cognitive restructuring and breathing training, interoceptive exposure, recording of panic symptoms, relapse prevention
Meyerbroeker et al., 2013	Gradual manipulation of crowd density in situations	nVisor SX: 1,280 × 1,024/60° (or CAVE with projection on three walls and floor^b^)	N/A	Supermarket, subway, Italian restaurant with bar annex, town center, large open square, marketplace with market stalls, public building with large open spaces and different floors with café on the ground floor; crowd density could be manipulated	Supermarket, shopping malls, marketplaces, streets and public transportation (e.g., subway)	Psychoeducation, cognitive restructuring, interoceptive exposure, discussion of safety behaviors, relapse prevention

*This table provides detailed information on the exposure treatment materials and procedures and on additional interventions applied in participants of the included studies. It mentions the general exposure strategy that was similar in VR and in vivo. Moreover, it gives information on the type of HMD used for visual stimuli presentation, including data on image resolution and field of view (FoV). The image resolution is reported by the number of pixels arranged horizontally and vertically; the field of view is reported as diagonal FoV in degrees. If available, information on the movement mode in VR and on additional devices for tactile stimulation is provided. The table furthermore provides descriptions of the VR and in vivo exposure environments and mentions psychological interventions that were applied in addition to pure exposure treatment in the VR as well as for the in vivo exposure condition. Studies are sorted by the type of phobia and date of publication. N/A: no information available*.

a *Seat with woofer under it to create noise and vibrations*.

b *In this study, a CAVE system was used in addition to HMD as an alternative mode for VR presentation. No significant effects of HMD vs. CAVE were found on outcome-measures*.

c *Imaginal exposure was conducted during in vivo exposure on a stationary plane*.

As presented in Table 1, the nine studies were published between 2000 and 2016 and included data from 371 participants overall, with a mean sample size of 41.22 patients (*SD* = 14.39). All studies included participants with the ICD or DSM diagnosis of a phobic anxiety disorder. In the two studies on flight phobia as a Specific Phobia, also patients with an Agoraphobia with flying as the main feared stimulus were included (Rothbaum et al., 2000, 2006). In one study on Agoraphobia (Botella et al., 2007), 17.1% of all participants including a waitlist condition were diagnosed with Panic Disorder without Agoraphobia. The study on spider phobia as a Specific Phobia included four participants with only a partial diagnosis of Specific Phobia who however scored within the phobic range for the questionnaire measures and behavioral avoidance task (Michaliszyn et al., 2010). The age of the included participants ranged from 18 to 72 - referring to those studies providing information on this sample characteristic (see Table 1). In all studies except one (Emmelkamp et al., 2002), more women than men were included, though one study did not give information on the distribution of sexes (see Table 1). Information on the percentage of medicated participants was only available in three studies (Botella et al., 2007; Michaliszyn et al., 2010; Bouchard et al., 2016) and showed a wide range of medication rates from zero to 66.6%. The number of total treatment sessions applied to each participant ranged from four (Emmelkamp et al., 2002) to 14 (Bouchard et al., 2016), with a mean total treatment session number of 8.78 (*SD* = 2.64). The number of total treatment sessions includes exposure sessions as well as additional sessions, for example with interventions like psychoeducation or relapse prevention (see Table 2). As required by the eligibility criteria of this meta-analysis (see section Eligibility Criteria), the amount of exposure was equal in the VR and in the *in vivo* exposure condition of all included studies. The amount of exposure was typically assessed by the number of exposure sessions, and the duration of one exposure session was also considered. In all studies, the number of exposure sessions performed in the VR and the *in vivo* group ranged from three sessions (Emmelkamp et al., 2002) to eight sessions (Bouchard et al., 2016) with a mean number of exposure sessions of 5.44 (*SD* = 1.59). The duration of one exposure session ranged from 20 to 90 min with a mean duration of 54.29 min (*SD* = 22.81) – though two studies did not give information on this treatment procedure characteristic (see Table 1).

As presented in Table 2, the exposure strategy for both the VR and *in vivo* group was described as gradual in all studies except one. This study mentioned a special feature of their exposure strategy, where the focus was to develop new, non-threatening and adaptive interpretations, and that habituation was not required (Bouchard et al., 2016). All studies applied a therapist guided exposure approach in the VR and *in vivo* condition. They all used HMD devices for visual stimuli presentation in the VR exposure condition. Image resolution and field of view of HMDs differed over the individual devices. The image resolution determines how clean the picture quality is, while the field of view (FoV) refers to the view or the surroundings that a human eye can see without eye movements (Jerdan et al., 2018). One study additionally used a CAVE system for visual stimuli presentation but did not find significant differences in contrast to an HMD presentation (Kampmann et al., 2016). For tactile and haptic stimulation, two studies on flight phobia mentioned the use of a specific seating construction in the VR condition (Rothbaum et al., 2000, 2006), and one study on fear of heights used a railing to hold on to (Emmelkamp et al., 2002). Some studies provided information on the movement mode in VR and mentioned either the use of a mouse (Botella et al., 2007; Michaliszyn et al., 2010; Bouchard et al., 2016), or that the participants could walk around freely in a demarcated space (Emmelkamp et al., 2002). Concerning the exposure environments for the VR and *in vivo* condition, some studies translated the *in vivo* environments directly into VR environments (Emmelkamp et al., 2002; Meyerbroeker et al., 2013), and others used *in vivo* environments that slightly differed from the VR environments. In one study on Social Phobia, *in vivo* group therapy was used to create a real-life audience for participants delivering a speech (Anderson et al., 2013). The study by Kampmann et al. (2016) provided standardized social scenarios in the VR condition but conducted exposure exercises on the participants' individual social situations in the *in vivo* condition. All included studies on Social Phobia furthermore mention the realization of social interactions with negative reactions of counterpart(s) in particular for the VR condition but not for the *in vivo* condition. Anderson et al. (2013) list bored, hostile and distracted as reactions of a virtual audience, Kampmann et al. (2016) mention dialogues with an unfriendly content, and - most pronounced - Bouchard et al. (2016) name acting under the scrutiny of strangers and facing criticism or insistence while meeting unfriendly neighbors or while refusing to buy from a persistent shop seller as virtual scenarios (see Table 2). In the two studies on fear of flying, VR and *in vivo* exposure differed in that way that no real flight was realized in the *in vivo* condition, but instead imaginal exposure of take-off, flight and a landing was conducted while sitting on a stationary plane (Rothbaum et al., 2000, 2006). In the study on spider phobia (Michaliszyn et al., 2010), *in vivo* exposure consisted of handling a living spider with the hands, while no tactile feedback was provided in VR. As additional interventions accompanying exposure, all studies conducted introduction interventions like psychoeducation. Most studies furthermore conducted cognitive or behavioral fear management strategies in addition to pure exposure treatment (Rothbaum et al., 2000, 2006; Botella et al., 2007; Michaliszyn et al., 2010; Meyerbroeker et al., 2013; Anderson et al., 2016; Bouchard et al., 2016). The two studies on Agoraphobia (Botella et al., 2007; Meyerbroeker et al., 2013) and the two studies on flight phobia (Rothbaum et al., 2000, 2006) applied interoceptive exposure in addition to VR and *in vivo* exposure. In all studies, additional interventions were conducted in the VR and the *in vivo* condition.

### Risk of Bias Within the Studies

To reduce the risk of bias within the included studies, only randomized-controlled trials and only studies on participants with valid diagnoses were selected. The main goal of this meta-analyses was to compare studies that applied an equal amount of exposure in the VR and *in vivo* condition, which is an important contribution to reduce the risk of bias. However, it should be noted that all studies were published by authors that are researchers in the field of VR exposure, which may enhance a particular risk of bias.

To assess common sources of bias within randomized-controlled trials in detail, we used a bias detection tool from the Cochrane Collaboration (Higgins et al., 2011). Altogether, our assessment largely showed a low to unclear risk of bias in the included studies (see Table 3). Concerning the risk of selection bias in particular, all studies used random assignment, but not all studies described the concrete procedure of random sequence generation and allocation concealment. Concretely, there was an unclear risk of selection bias in six studies, as the method of random sequence generation and allocation concealment was not further specified. One study reported an assignment based on a computerized random number generator and the participation of a third study coordinator to ensure an unknown allocation before the participants' enrollment (Anderson et al., 2013), one study reported an assignment based on random number tables and a concealed assignment not further specified (Bouchard et al., 2016), and one study reported the use of a computerized random number generator and a concealed assignment using envelopes prepared by a third person and opened after enrollment of the participants (Kampmann et al., 2016). All of the above had a low selection bias risk. A performance bias must be suspected in all studies, as the blinding of participants and researchers during VR or, respectively, *in vivo* exposure was not possible due to the nature of the intervention. Generally, not all sources of bias can be avoided, due to the nature of the applied intervention. A certain performance bias therefore must be tolerated. Nevertheless, at least one study reported the blinding of the participant and researcher until the exposure component was applied, thereby enabling blind pre-processing interventions (Botella et al., 2007). This procedure was rated as low performance bias risk, considering the nature of the treatment. Other studies did not further specify the time point of de-blinding and therefore the risk of performance bias concerning pre-processing remained unclear. Also blinding of outcome assessment was not entirely realizable as the meta-analysis was conducted on self-report measurements of phobic fear and participants could therefore not be blind to the applied condition at post measurement. Though, two studies realized blinding during pre-assessment as an approximation (Botella et al., 2007; Kampmann et al., 2016), both rated as low detection bias risk under the decribed circumstances. Other studies did not further specify if pre-processing was performed blind and were rated as an unclear risk of detection bias. Risk of attrition bias was low in many studies; however, some studies did not provide sufficient information, therefore, risk of attrition bias remained unclear. In studies with an intent-to-treat data, attrition bias was rated as low, if losses to post-test were disclosed with respective reasons, intent-to-treat analysis method was described, and if means and standard deviations were reported with information on the sample size in both groups (Meyerbroeker et al., 2013; Bouchard et al., 2016). If those descriptions were incomplete in studies with an intent-to-treat sample, attrition bias risk was rated as unclear. One study, where the completers and intent-to-treat sample were the same and outcome data therefore was complete (Botella et al., 2007), was rated as having a low risk of attrition bias. In studies with data for the completer sample, risk of attrition bias was rated as low if a precise description of attrition and exclusion of patients was provided (Rothbaum et al., 2000; Emmelkamp et al., 2002). In comparison, in studies with participants switching between conditions at an unspecified point of time (Michaliszyn et al., 2010), risk of attrition bias was rated as high. Risk of reporting bias was rated as low in all studies, as prespecified outcome measures were all reported.

**Table 3:** Assessment of risk of bias within the studies.

	**Risk of selection bias**		**Risk of performance bias**	**Risk of detection bias**	**Risk of attrition bias**	**Risk of reporting bias**
**References**	**Random sequence generation**	**Allocation concealment**	**Blinding of participants and researchers**	**Blinding of outcome assessment**	**Incomplete outcome data**	**Selective reporting**
Rothbaum et al., 2000	Unclear	Unclear	Unclear	Unclear	Low	Low
Emmelkamp et al., 2002	Unclear	Unclear	Unclear	Unclear	Low	Low
Rothbaum et al., 2006	Unclear	Unclear	Unclear	Unclear	Unclear	Low
Michaliszyn et al., 2010	Unclear	Unclear	Unclear	Unclear	High	Low
Anderson et al., 2013	Low	Low	Unclear	Unclear	Unclear	Low
Kampmann et al., 2016	Low	Low	Unclear	Low	Unclear	Low
Bouchard et al., 2016	Low	Low	Unclear	Unclear	Low	Low
Botella et al., 2007	Unclear	Unclear	Low	Low	Low	Low
Meyerbroeker et al., 2013	Unclear	Unclear	Unclear	Unclear	Low	Low

*Risk of bias was assessed using a tool from the Cochrane Collaboration (Higgins et al., 2011). Risk of bias was rated as low, unclear, or high*.

### Results of Individual Studies

Table 4 shows means and standard deviations of the anxiety measures at pre and post assessment, as well as sepereate Hedges' *g* effect sizes for pre-post treatment effects for both the VR and *in vivo* group. Effect sizes for the VR exposure condition ranged from 0.35 (Rothbaum et al., 2006) to 2.76 (Michaliszyn et al., 2010), while effect sizes for the *in vivo* exposure condition ranged from 0.31 (Rothbaum et al., 2006) to 3.86 (Michaliszyn et al., 2010). Six studies conducted an intent-to-treat analysis (Rothbaum et al., 2006; Botella et al., 2007; Anderson et al., 2013; Meyerbroeker et al., 2013; Bouchard et al., 2016; Kampmann et al., 2016), one of them reporting the same sample size for participants included in the study and completers (Botella et al., 2007). Three studies reported on the completer sample (Rothbaum et al., 2000; Emmelkamp et al., 2002; Michaliszyn et al., 2010).

**Table 4:** Effect sizes for the pre-post treatment effects of VR exposure therapy and *in vivo* exposure therapy.

						**Pre**		**Post**		**Effect size**		
											**95% CI**	
**References**	**Phobia**	**Sample**	**Anxiety measure**		***n***	***M***	***SD***	***M***	***SD***	***g***	**LL**	**UL**
Rothbaum et al., 2000	Specific Phobia	Completer	FFI	VRET	15	105.85	35.91	86.14	37.40	0.51	−0.22	1.23
	(fear of flying)			IVET	15	133.30	42.00	87.53	42.30	1.03	0.17	1.88
Emmelkamp et al., 2002	Specific Phobia	Completer	AQ-Anxiety	VRET	17	57.12	12.18	36.12	20.56	1.18	0.33	2.04
	(fear of heights)			IVET	16	59.06	17.12	42.19	17.14	0.93	0.13	1.74
Rothbaum et al., 2006	Specific Phobia	ITT	FFI	VRET	29	120.38	44.24	103.69	49.35	0.35	−0.17	0.86
	(fear of flying)			IVET	29	116.79	57.74	100.34	43.49	0.31	−0.20	0.83
Michaliszyn et al., 2010	Specific Phobia (fear of spiders)	Completer^a^^,^ ^b^	FSQ	VRET	16	104.61	9.59	54.37	22.46	2.76	1.26	4.27
				IVET	16	103.28	13.13	47.88	14.07	3.86	1.86	5.87
Anderson et al., 2013	Social Phobia	ITT	PRCS	VRET	30	24.37	2.54	16.23	7.61	1.40	0.70	2.10
				IVET	39	25.59	2.59	14.79	8.53	1.68	1.00	2.36
Kampmann et al., 2016	Social Phobia	ITT^a^	LSAS-SR	VRET	20	73.00	17.25	55.74	18.65	0.92	0.20	1.64
				IVET	20	69.15	19.44	39.22	25.01	1.28	0.46	2.10
Bouchard et al., 2016	Social Phobia	ITT	LSAS-SR	VRET	17	85.1	29.5	51.8	23.3	1.19	0.34	2.05
				IVET	22	74.9	24.5	56.0	26.9	0.71	0.07	1.35
Botella et al., 2007	Agoraphobia	Completer = ITT^c^	FQ-Agoraphobia	VRET	12	16.27	14.19	6.82	7.61	0.77	−0.09	1.64
				IVET	12	14.58	11.80	4.25	6.35	1.01	0.07	1.95
Meyerbroeker et al., 2013	Agoraphobia	ITT^b^	ACQ	VRET	24	2.58	0.52	1.96	0.53	1.14	0.43	1.85
				IVET	22	2.63	0.66	2.02	0.74	0.84	0.17	1.51

*This table provides means and standard deviations of pre and post measurements on the stated anxiety measures, as well as pre to post effect sizes for VR exposure therapy (VRET) and in vivo exposure therapy (IVET) of all studies included in the meta-analysis. Effect sizes were reported as Hedges' g. The statistical values either refer to the completer sample (Completer) or to the intent-to-treat sample (ITT), as mentioned. Studies are sorted by the type of phobia and date of publication. AQ-Anxiety, Acrophobia Questionnaire, Anxiety-subscale; FSQ, Fear of Spiders Questionnaire; FFI, Fear of Flying Inventory; PRCS, Personal Report of Confidence as a Speaker; LSAS-SR, Liebowitz Social Anxiety Scale; FQ-Agoraphobia, Fear Questionnaire – Agoraphobia; ACQ, Agoraphobic Cognition Questionnaire; CI, confidence interval; LL, lower limit; UL, upper limit*.

a *The report did not present sample sizes and/or a declaration of ITT or completer sample in the table on means and standard deviations, information from the text was used for specification*.

b *Patients from waitlists were allocated to the VRET and IVET condition and included in the analysis*.

c *The authors reported the same sample size for the number of participants included in the study and the analysis sample*.

**Figure 2:**
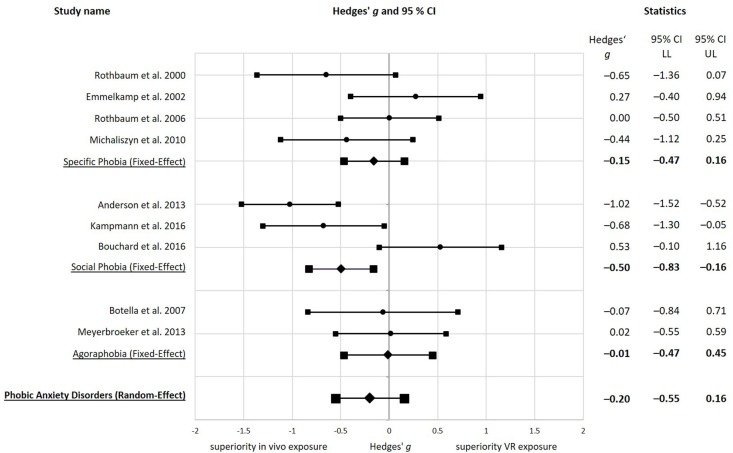
Forest plot with pre to post effect sizes for the comparison of VR exposure therapy to *in vivo* exposure therapy. All effect sizes are reported as Hedges' *g*, using a fixed-effect model or a random-effect model as stated. Negative effect sizes indicate superiority of *in vivo* exposure therapy, while positive effect sizes indicate superiority of virtual reality exposure therapy. Studies are sorted by the type of phobia and date of publication.

In the comparison of the treatment effects of the VR exposure and the *in vivo* exposure condition in the individual studies (see Figure 2), one study showed a large (*g* ≥ 0.80) (Anderson et al., 2013), two studies a medium (0.80 > *g* ≥ 0.50) (Rothbaum et al., 2000; Kampmann et al., 2016) and one study a small (0.50 > *g* ≥ 0.20) (Michaliszyn et al., 2010) negative effect size, indicating superiority of *in vivo* exposure. One study showed a small (0.50 > *g* ≥ 0.20) (Emmelkamp et al., 2002) and one study a medium (0.80 > *g* ≥ 0.50) (Bouchard et al., 2016) positive effect size in the direction of superiority of VR exposure over *in vivo* exposure therapy. Three studies showed an effect size around zero (−0.06 to 0.02) and thereby below a small effect (*g* < 0.20) (Rothbaum et al., 2006; Botella et al., 2007; Meyerbroeker et al., 2013), pointing to no relevant difference between VR and *in vivo* exposure therapy.

### Synthesized Findings

Both, Virtual Reality exposure therapy (*g* = 1.00) and *in vivo* exposure therapy (*g* = 1.07) showed a large, significant overall effect size, when synthesizing the nine included studies (*n* = 371) on phobic anxiety disorders using a random-effect model (see Table 5). Calculated separately for each phobic anxiety disorder using fixed-effect models, VR exposure therapy showed a medium, significant effect size in Specific Phobia (*g* = 0.68), and a large, significant effect size in Social Phobia (*g* = 1.17) and Agoraphobia (*g* = 0.99). *in vivo* exposure therapy also yielded a medium, significant effect size in Specific Phobia (*g* = 0.72), and a large, significant effect size in Social Phobia (*g* = 1.19) and Agoraphobia (*g* = 0.90) (see Table 5).

**Table 5:** Pooled effect sizes for the pre-post-treatment effects of VR exposure therapy and *in vivo* exposure therapy.

	**VR exposure**						***In vivo*** **exposure**				
					**95%CI**					**95% CI**	
**Synthesis (Model; number of pooled studies)**	***n***	***g***	***SEg***	***p***	**LL**	**UL**	***g***	***SEg***	***p***	**LL**	**UL**
Specific Phobia (Fixed-Effect; *n* = 4)	153	0.68	0.22	<0.001	0.32	1.05	0.72	0.19	<0.001	0.34	1.10
Social Phobia (Fixed-Effect; *n* = 3)	148	1.17	0.22	<0.001	0.74	1.61	1.19	0.21	<0.001	0.79	1.60
Agoraphobia (Fixed-Effect; *n* = 2)	70	0.99	0.28	<0.001	0.44	1.54	0.90	0.28	0.001	0.35	1.44
All Phobic Anxiety Disorders (Random-Effect; *n* = 9)	371	1.00	0.18	<0.001	0.65	1.35	1.07	0.21	<0.001	0.66	1.47

*This table presents pre to post effect sizes for VR exposure therapy and for in vivo exposure therapy pooled from studies on Specific Phobia, Social Phobia, Agoraphobia, and all studies. Pooled effect sizes are reported as Hedges' g using a fixed-effect model or random-effect model as stated. CI, confidence interval; LL, lower limit; UL, upper limit*.

For the comparison of the treatment effect of VR exposure therapy and *in vivo* exposure therapy in all nine included studies on phobic anxiety disorders (*n* = 371), using a Hedges' *g* random-effects model, we obtained a mean overall effect size estimate of Hedges' *g* = −0.20, *SE* = 0.18, *p* = 0.271, 95% CI [−0.55, 0.16] (see Figure 2). The negative effect size represents a difference of mean treatment changes in the direction of superiority of *in vivo* exposure, but the effect size was at the lower limit of a small effect (0.50 > *g* ≥ 0.20) and therefore very small, and not significantly different from zero. Accordingly, we found no evidence for a significant difference in the efficacy of VR and *in vivo* exposure therapy over all studies on phobic anxiety disorders.

To separately compare the treatment effects of VR exposure and *in vivo* exposure therapy for the three subtypes of phobic anxiety disorders, we applied fixed-effect models (see results in Figure 2), which are appropriate for the small number of studies included on each phobia. The pooled effect size for four studies on Specific Phobia (*n* = 153) showed a non-significant result in favor of *in vivo* exposure that was below the level of a small effect (*g* < 0.20), *g* = −0.15, *SE* = 0.16, *p* = 0.333, 95% CI [−0.47, 0.16]. This means that we found no significant difference in the efficacy of VR exposure and *in vivo* exposure in Specific Phobia. The pooled effect size of three studies on Social Phobia (*n* = 148) showed a medium and significant effect size favoring *in vivo* exposure, *g* = −0.50, *SE* = 0.17, *p* = 0.003, 95% CI [−0.83, −0.16]. Accordingly, VR exposure therapy was found to be significantly less efficacious than *in vivo* exposure therapy in Social Phobia. The pooled effect size for two studies on Agoraphobia (*n* = 70) yielded a non-significant result close to zero, *g* = −0.01, *SE* = 0.23, *p* = 0.959, 95% CI [−0.47, 0.45], not favoring one of the treatment conditions. This indicates similar treatment effects of VR and *in vivo* exposure therapy in Agoraphobia.

### Risk of Bias

**Figure 3:**
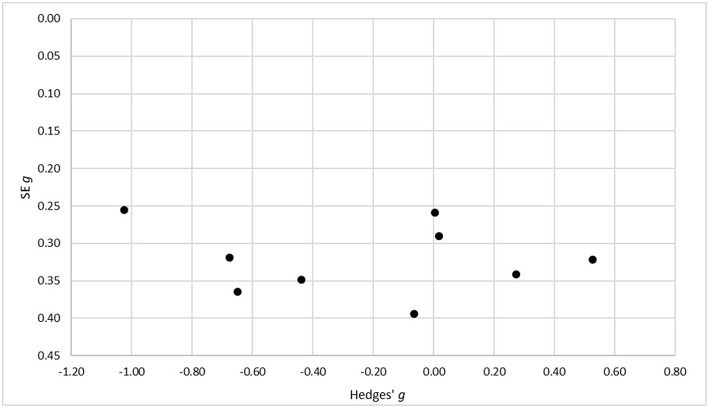
Funnel Plot for the detection of publication bias across studies with Hedges' *g* on the *x*-axis and standard errors for Hedges' *g* on the *y*-axis.

Visual inspection of a funnel plot (see Figure 3) showed a sample of studies with relatively homogenous standard errors and widespread effect sizes. There was no asymmetry detected indicating a (publication) bias.

## Discussion

### Summary and Discussion of Main Findings

Applying strict inclusion criteria to focus exclusively on the comparison of VR and *in vivo* exposure, this meta-analysis synthesized nine randomized-controlled trials with altogether 371 participants, comparing the pre to post treatment effects of VR and *in vivo* exposure therapy in phobic anxiety disorders applied with an equivalent amount of exposure and with equivalent additional interventions alongside exposure in both conditions. VR and *in vivo* exposure both yielded large effect sizes concerning the reduction of phobic fear. For the comparison of VR and *in vivo* exposure therapy, we found a small, but non-significant effect size (*g* = −0.20, *p* = 0.271) favoring *in vivo* exposure over VR exposure (see Figure 2). Although a non-significant effect is not a final proof of equivalence, it shows that there is no evidence that VR exposure is significantly less efficacious than *in vivo* exposure therapy in phobic anxiety disorders. The 95% confidence interval of the synthesized effect size ranged from −0.55 to 0.16. This illustrates that the true effect may lie in this range and that VR exposure could be inferior to slightly superior in comparison to *in vivo* exposure. Regarding previous meta-analyses on the comparison of VR to *in vivo* exposure, a non-significant effect size is consistent with the finding of a recent meta-analysis on VR versus *in vivo* exposure in anxiety disorders by Carl et al. (2019), which showed a non-significant, negative effect size in favor of *in vivo* exposure (*g* = −0.07, *p* = 0.544). However, this effect size was even below the level of a small effect. An earlier meta-analysis by Powers and Emmelkamp (2008) even reported a small, positive effect size in favor of VR exposure over *in vivo* exposure therapy (*g* = 0.34). Those differences of our results to former meta-analyses comparing VR to *in vivo* exposure might be due to factors like the number and type of studies included, the selected outcome measures, or the data analysis strategy. Powers and Emmelkamp (2008) included only five studies published until 2007 and in this regard conducted their meta-analysis on a smaller sample of original studies. Carl et al. (2019) synthesized 14 studies, and included studies with a different amount of exposure between conditions (Pelissolo et al., 2012), with imaginal instead of *in vivo* exposure in the control group (Wallach et al., 2009), with *in vivo* exposure only for patients with comorbid conditions in the control group (Krijn et al., 2007), with VR presentation without using immersive systems (e.g., HMD) and head tracking (Klinger et al., 2005), as well as preliminary data (Robillard et al., 2010) on an already included study (Bouchard et al., 2016). These studies were excluded in our meta-analysis due to the stricter inclusion criteria. As one important point, we ensured that *in vivo* exposure was applied for all clients in the control condition and in an equivalent amount to VR exposure. That might have made the control condition more powerful thereby shifting our overall effect size toward the superiority of *in vivo* exposure in comparison to the previous meta-analysis. Furthermore, we diminished potential sources of bias like dependent samples. For example, the study consisting of preliminary data showed a medium positive effect size in favor of VR exposure, whereby the inclusion of this study in the meta-analysis by Carl et al. (2019) might have moved their overall effect size toward VR exposure.

Considering the results for the individual studies included in our meta-analysis, the effect sizes for the pre to post treatment efficacy of VR in comparison to *in vivo* exposure therapy varied largely (−1.02 to 0.53) (see Figure 2), with some favoring VR exposure, some favoring *in vivo* exposure and some detecting no relevant differences. The wide range shows the high potential of VR, but also illustrates that VR exposure therapy could be less efficacious than *in vivo* exposure therapy. This raises a discussion on potential working mechanisms of a more or less efficacious VR exposure therapy if compared to *in vivo* exposure. On the one hand, variance in the effect sizes of the individual studies might be due to confounding variables, like differences in the distribution of participants' characteristics for the two conditions (e.g., age, comorbidities, or severity of phobic anxiety). On the other hand, the variance could result from differences in the specific materials and procedures of exposure therapy in the individual studies (e.g., technical features of VR devices, realization of VR and *in vivo* environments, or combination of exposure with accompanying treatment elements, for example cognitive interventions). Furthermore, it could be due to differences in the efficacy of VR exposure for different kinds of phobic anxiety disorders. We discuss these factors in the paragraphs that follow.

Looking separately at studies on Specific Phobia (*n* = 4), we found a negative effect size in favor of *in vivo* exposure (*g* = −0.15) (see Figure 2), which was however non-significant and furthermore below the level of a small effect. VR exposure consequently does not seem to be significantly less efficacious than *in vivo* exposure in this phobic anxiety disorder. This is consistent with the results of the latest meta-analysis on VR versus *in vivo* exposure in anxiety disorders by Carl et al. (2019), who particularly synthesized five studies comparing VR and *in vivo* exposure in Specific Phobia. Other than Carl et al. (2019), the meta-analysis conducted here, excluded a study with *in vivo* exposure only for patients with comorbid conditions in the control group (Krijn et al., 2007), and thereby could prove the result for a sample of studies free from understated *in vivo* conditions.

Behind the synthesized effect size of four studies on Specific Phobia, the effect sizes of the individual studies ranged from −0.65 to 0.27 (see Figure 2). It is important to mention that we pooled studies on three different Specific Phobias (fear of heights, fear of spiders and fear of flying) as there were not enough articles published to synthesize results separately for each Specific Phobia. Effect sizes indicating superiority of *in vivo* exposure were found for two studies, one on fear of spiders (Michaliszyn et al., 2010) and one on fear of flying (Rothbaum et al., 2000). Michaliszyn et al. (2010) assumed that inferiority of VR exposure might be based on an insufficient presence or problems with cybersickness. Another aspect to consider is that *in vivo* exposure was defined as successful in this study once patients could handle a living spider in their hand (Michaliszyn et al., 2010). In contrast, VR exposure did not consist of tactile stimulation (see Table 2) which could have diminished its efficacy when compared to *in vivo* exposure. Another point to explain inferiority of VR in one study conducted earlier in time (Rothbaum et al., 2000) might be the use of an older HMD technology. Against this hypothesis speaks a comparison between the two studies on fear of flying conducted by the group around Barabara Rothbaum (Rothbaum et al., 2000, 2006) with a relative similar treatment proecedure. The study conducted 2006 pointed more toward equivalency of VR and *in vivo* exposure than the earlier study conducted 2000, although an HMD with an equal amount of pixel and a lower field of view was used in the 2006 study (see Table 2). Therefore, further potential (confounding) variables, for example differences in the sample characteristics between both studies, must be considered as relevant for the difference in effect sizes. Another discussion point concerning those two studies is, that Rothbaum et al. (2000) and Rothbaum et al. (2006) both conducted an *in vivo* exposure of a parked plane but only imaginal exposure of a takeoff, flying, and landing in the control group. In contrast, VR exposure consisted of a takeoff, landing, and flying in calm and stormy weather (see Table 2). Therefore, a flight exposure conducted entirely *in vivo* might still yield superior effects in comparison to VR exposure. Hence, the comparison of VR and *in vivo* flight exposure is an interesting question for future research. The study on fear of heights by Emmelkamp et al. (2002), showing a small effect size favoring VR therapy, indicates that VR exposure can be equally efficacious or even superior than *in vivo* exposure. In this study, participants in the VR group were exposed to exactly the same three situations as participants in the *in vivo* group, which were rebuilt as VR environments (see Table 2). Furthermore, participants could walk around freely in a demarcated space during VR exposure in this study, while other studies like for example Michaliszyn et al. (2010) used a mouse for movements in VR (see Table 2). This could potentially represent an advantage of the VR condition in this study when compared to the VR conditions of other studies.

The result for studies on Agoraphobia (*n* = 2) distinctly points to a similar efficacy of VR and *in vivo* exposure, as the synthesized effect size is close to zero and non-significant (*g* = −0.01) (see Figure 2). Here, the separate effect sizes for the individual studies were homogeneous, ranging from −0.07 to 0.02. In both included studies, participants were gradually exposed to situations like a subway, public buildings, or supermarkets in VR, and a similar *in vivo* exposure was applied in the control group (Botella et al., 2007; Meyerbroeker et al., 2013) (see Table 2). Unlike our result, the meta-analysis on VR versus *in vivo* exposure in anxiety disorders by Carl et al. (2019) synthesized three studies on Panic Disorder with Agoraphobia and found a small, negative effect size in favor of *in vivo* exposure (g = −0.26), which was, however, non-significant. Carl et al. (2019) additionally included one study that performed CBT methods classically recommended for panic disorder (among them *in vivo* exposure) in the control group, and VR exposure to different environments as the main intervention in the experimental group (Pelissolo et al., 2012). This study yielded a negative effect size in favouring of *in vivo* exposure, which might possibly be due to the application of interventions relevant for panic disorder patients such as relaxation training, cognitive interventions, and interoceptive exposure in the *in vivo* but not in the VR condition. The study was excluded from our meta-analysis due to the stricter eligibility criteria of an equivalent amount of exposure and the equivalency of additional interventions in the VR and *in vivo* condition. Its negative effect size probably explains the stronger trend in favor of *in vivo* exposure for Agoraphobia in the meta-analysis by Carl et al. (2019). The two studies included in our meta-analysis on Agoraphobia conducted cognitive interventions and interoceptive exposure as additional interventions in both the VR and *in vivo* exposure group (Botella et al., 2007; Meyerbroeker et al., 2013) (see Table 2). Considering this, we suspect that VR and *in vivo* exposure in Agoraphobia show a similar efficacy in studies with a highly comparable treatment procedure of exposure and additional interventions in VR and *in vivo* therapy.

Interestingly, we found a significant, medium effect size (*g* = −0.50) in favor of *in vivo* exposure in the synthesis of studies comparing VR and *in vivo* exposure in Social Phobia (*n* = 3) (see Figure 2). This indicates evidence for the superiority of *in vivo* exposure in Social Phobia and therefore represents an inconsistency to former meta-analyses. So, Chesham et al. (2018) found an extremely small, non-significant effect in favor of VR exposure when pooling five randomized trials comparing VR exposure with standard treatment (*in vivo* or imaginal exposure) for Social Anxiety. Also, Carl et al. (2019) found a small, non-significant effect in favor of VR exposure when comparing to *in vivo* exposure in six studies on Social Anxiety and Performance Anxiety. Unlike Carl et al. (2019) and Chesham et al. (2018), we excluded studies with imaginal exposure or with a video-taped visualization procedure but with no *in vivo* exposure as the control group (Wallach et al., 2009; Heuett and Heuett, 2011), because the abscence of *in vivo* exposure could lower the efficacy of the control condition in contrast to the VR condition. Both studies actually yielded either a positive effect size in favor for VR exposure (Heuett and Heuett, 2011) or an effect size close to zero, while not favoring VR or *in vivo* exposure (Wallach et al., 2009). Moreover, unlike Carl et al. (2019) we excluded studies with VR presentation without using immersive systems (e.g., HMD) and head tracking (Klinger et al., 2005), and studies on preliminary data on already included trials (Robillard et al., 2010), that both yielded positive effect sizes in favor of VR. The exclusion of those four studies in our meta-analysis can probably explain why our results differ from the meta-analysis by Carl et al. (2019) or Chesham et al. (2018).

We found a superiority of *in vivo* exposure over VR exposure only in Social Phobia but not in Agoraphobia and Specific Phobia, indicating that it might be more difficult to create virtual social environments for Social Phobia exposure than virtual spiders, heights and airplanes for Specific Phobia exposure, or virtual shopping malls, subways, or tunnels for Agoraphobia exposure. In addition, Social Phobia is considered to be a more complex disorder with high comorbidity, chronicity, and impairment (Wittchen and Fehm, 2003), and shows lower remission rates in CBT treatments than other anxiety disorders (Springer et al., 2018). Actually, these issues should affect VR as well as *in vivo* exposure therapy in Social Phobia. Nevertheless, it might be easier to activate specific dysfunctional beliefs of Social Phobia patients (e.g., concerning what others think of them) *in vivo* than in VR. In general, the creation of avatars, agents, and social interactions for VR settings is an issue which is challenging not only for psychological but also for computer science research. As an example, the degree of naturalism required for virtual agents is being intensively discussed, which seems to not be linearly related to the users' acceptance of the agent (“uncanny valley effect”) (see for e.g., Stein and Ohler, 2017; Schwind et al., 2018).

The effect sizes on the comparison of VR to *in vivo* exposure from the individual reports on Social Phobia ranged from −1.02 to 0.53 (see Figure 2). This shows that VR exposure was partially inferior and partially superior to *in vivo* exposure in the studies included in our meta-analysis. An effect size favoring *in vivo* exposure was found in the study on public speaking phobia by Anderson et al. (2013) in which participants were asked to deliver a speech in front of a virtual audience varying in size, if assigned to the VR condition, or respectively, in front of a real audience in a group therapy setting if allocated to the *in vivo* group (Anderson et al., 2013) (see Table 2). In VR, the audience members could be manipulated on their reactions (e.g., bored or interested; see Table 2) and could pose standardized questions (Anderson et al., 2013). As an important difference, participants in the *in vivo* group not only delivered their own speech but additionally listened to the speeches of other group therapy members and received positive feedback on their own videotaped speeches from the whole group instead of only from the therapist like realized in the VR condition (Anderson et al., 2013) (see Table 2). According to the authors of the study, the group setting might have been of a stronger interpersonal nature than the VR environment. Furthermore, they considered the feedback as a higher dose of exposure in the *in vivo* condition (Anderson et al., 2013). Above that, one might speculate that the *in vivo* condition provided a higher individualization in social interactions in contrast to the standardized reactions and questions of the virtual audience in the VR condition. Furthermore, positive feedback from numerous peers might have supported cognitive reinterpretations of the feared situation and observing speeches from other participants could have possibly worked as model learning. This might represent advantages of the *in vivo* in contrast to the VR condition. In the second study yielding an effect size in favor of *in vivo* exposure (Kampmann et al., 2016), a gradual exposure to different standardized social situations with standardized dialogues with different content and style from friendly to unfriendly and with varying personal relevance was conducted in the VR group, whereas individual social situations were translated to *in vivo* exposure exercises in the *in vivo* control group, and also exposure in the personal environment of the participants was realized in this condition (see Table 2). Both conditions were not combined with cognitive interventions to examine the pure effect of exposure therapy (Kampmann et al., 2016). It might be possible that a stronger individualization of the situations in the *in vivo* group resulted in a higher efficacy of *in vivo* in comparison to VR exposure. The authors of the study furthermore speculated that the results could be attributed to the fact that it was their first version of a Social Anxiety VR environment, and moreover mentioned that VR exposure for Social Phobia might need to be combined with cognitive elements to improve efficacy (Kampmann et al., 2016). Superiority of VR exposure in Social Phobia was actually achieved in a study which realized social situations in VR and *in vivo* while focusing on cognitive restructuring without requiring habituation (Bouchard et al., 2016) (see Table 2). The authors of the study pointed out that cognitive interventions might influence the way exposure is mentally processed by the patients. As a further difference to the study conducted by Kampmann et al. (2016), the therapist was present in the same room during VR exposure. Bouchard et al. (2016) in this regard considered a better therapeutic alliance in the VR condition as another possible explanation for the different results. As a difference to the studies conducted by Anderson et al. (2013) and Kampmann et al. (2016), Bouchard et al. (2016) partially used role-playing for exposure in the *in vivo* condition. One might argue that role-play causes less fear activation than social situations in real-life, which could have lowered the efficacy of the *in vivo* in comparison to the VR condition of this study. Furthermore, it is to mention that the social situations realized in the *in vivo* condition of the study by Bouchard et al. (2016) seem to be less individualized in comparison to the study conducted by Kampmann et al. (2016). Finally, in the study by Bouchard et al. (2016) VR exposure scenarios included social interactions like acting under the scrutiny of strangers, being refused, or facing criticism or insistence that were not described for the *in vivo* condition (see Table 2). Although negative reactions of virtual counterparts were also realized in the studies by Anderson et al. (2013) and Kampmann et al. (2016) (see Table 2), they seem more pronounced in the study by Bouchard et al. (2016). Because negative reactions of a counterpart target central fears of Social Phobic patients, this might - especially when combined with cognitive interventions - for example facilitate expectancy violation concerning catastrophic beliefs. This could be a further aspect explaining why VR exposure was more efficacious than *in vivo* exposure in this study. Overall, real humans' reactions cannot be manipulated in the same systematical way as in VR, and social interactions comprising rejection are therefore not easily realized *in vivo*. This might generally represent an advantage of VR over *in vivo* exposure in Social Phobia.

To further discuss variables that might have influenced the efficacy of VR in comparison to *in vivo* exposure therapy, differences in the participants' characteristics, in the technical features of VR devices, and in the concrete kind of VR and *in vivo* exposure environments and procedures are considered.

As one sample characteristic, we looked at differences in the participants' age in all included studies. Age ranged from 18 to 72 years, with a mean age over both experimental groups ranging from 29.1 to 43.97 years (see Table 1). Although the variance was not strikingly high, differences in the participants' age between the individual studies might have had an influence on the efficacy of the VR exposure treatment for example. One might hypothesize that younger participants profit more from a VR treatment than older participants, as they are more familiar with this technique. Contradictory to this hypothesis, VR exposure was inferior to *in vivo* exposure (*g* = −0.44) in the study which included the youngest participants (age *M* = 29.1, *SD* = 7.99) (Michaliszyn et al., 2010). Furthermore, the study which included the oldest participants (age *M* = 43.97, *SD* = 9.34) showed a result in favor of VR exposure therapy (*g* = 0.27) (Emmelkamp et al., 2002). And also over all studies, visually inspection does not show a distinct positive relationship between the efficacy of VR in comparison to *in vivo* exposure and the mean age of the participants. Further sample characteristics like disorder severity, comorbidities, or medication for example, could not systematically be examined, as they have not been measured homogeneously in the different studies.

Another point to consider is the changes VR technologies experienced from 2000 to 2016, during the period in which the included studies were published. One might argue that the technical development of VR devices could have affected the therapy results in the VR exposure condition, while the *in vivo* exposure procedure stayed relatively unchanged. In this regard, better developed VR techniques might have led to higher efficacy of VR exposure treatments in comparison to *in vivo* exposure. As already mentioned in the discussion on effect sizes for Specific Phobia, the results for the studies by Rothbaum et al. (2000, 2006) do not point to this hypothesis. For a deeper examination, we provide a description of the resolution and field of view of the different types of HMDs used in the individual studies included in this meta-analysis (see Table 2). Over all studies, visual inspection does not show a positive relationship between the efficacy of VR in comparison to *in vivo* exposure and the technical development of the HMD devices. For example, VR exposure conducted with a nVisor SX, as a VR device with a - for the first decade of the century- relatively high resolution and a wide field of view (1280 × 1024/60°), in one case shows a similar efficacy of VR to *in vivo* exposure (g = 0.02) (Meyerbroeker et al., 2013), and an inferior efficacy of VR in another case (g = −0.68) (Kampmann et al., 2016). Moreover, studies using a VFX, as a type of HMD with a lower resolution and field of view (640 × 480/35°), also point at similarity between VR and *in vivo* exposure (Rothbaum et al., 2006), or show inferiority of VR exposure (Anderson et al., 2013). Studies conducted with a V6, as another type of HMD (640 × 480/60°), point at similarity between VR and *in vivo* exposure (g = −0.07) (Botella et al., 2007), or show an effect size in favor of *in vivo* exposure (g = −0.65) (Rothbaum et al., 2000).

Finally, variables like age and technical development of HMDs alone cannot explain differences in the efficacy of VR in comparison to *in vivo* exposure therapy in all the studies included in this meta-analysis. Instead, different modes of movement in VR, individualization of VR scenarios, the therapeutic alliance in VR exposure, the combination of VR exposure with cognitive interventions, and the creation of virtual social interactions targeting central fears are interesting factors to be considered in future research on the effective factors of VR exposure therapy, especially in Social Phobia. As soon as more studies are available, systematic meta-regression analysis could statistically examine the influence of certain variables on the efficacy of VR in comparison to *in vivo* exposure therapy. Therefore, original studies should not only control for participants' characteristics characteristics such as disorder severity, comorbidity, or accompanying medication as potential confounding variables, but also systematically describe their materials and procedure in matters of the VR settings such as movement mode in VR, stimulation of further senses alongside the sense of sight, design of virtual social interactions, exposure strategy, role of the therapist during exposure, as well as realization of theoretical and empirical approaches such as cognitive restructuring and inhibitory learning. Moreover, experimental studies could systematically variegate potential effective factors thereby providing findings on a causal influence on the efficacy of VR exposure. By doing this, future studies could reveal more about efficacious application procedures of VR exposure therapy.

### Limitations

The main limitation of this meta-analysis is the relatively small number of included studies, as only nine published articles fulfilled the eligibility criteria. This limits the generalizability of the results, especially for the specific phobic anxiety disorders. On the other hand, a statistical summary, even of a small number of studies, can be meaningful since it prevents intuitive *ad hoc* summaries which are often highly misleading (Borenstein et al., 2009). However, the relatively strict inclusion criteria of this meta-analysis for the participants, procedures, materials, and study design resulted in pooled effect sizes from a sample of highly comparable studies with a study methodology that was of comparatively good quality, but which could have been improved on.

As one eligibility criterion, we only included studies of participants diagnosed with an ICD or DSM phobic anxiety disorder, resulting in a relatively homogeneous sample of all studies. However, the inclusion of studies on three different phobic anxiety disorders resulted in a certain variance between participants. Moreover, there were studies which included participants diagnosed with other (phobic) anxiety disorders than the target disorder of the particular study. Those were, in particular, patients with Agoraphobia in the two studies on fear of flying (Rothbaum et al., 2000, 2006) and participants with Panic Disorder without Agoraphobia in a study on Agoraphobia (Botella et al., 2007) (see Table 1), which could cause a certain bias if those participants were not equally distributed in the experimental conditions.

Another limitation lies in the use of different anxiety measurements across different studies, which could have biased the synthesized effect sizes. As we calculated effect sizes on symptom specific anxiety measurements, it was necessary to pool different assessments for Specific Phobia, Social Phobia and Agoraphobia, but also, studies on the same phobic anxiety disorder applied different symptom specific measurement (see Table 4). Moreover, we only included self-report measurements, as there were not enough studies which conducted comparable objective measures, like behavioral avoidance tasks. Though, as standardized test were used, those should have generated sufficiently reliable and valid measurements.

Regarding VR equipment, only studies using VR devices with immersive systems (e.g., HMD) and head tracking were included, leading to a relatively homogeneous application of VR stimuli. Nevertheless, the studies did not all use the same type of hardware, such as different types of HMDs (see Table 2) and different types of software as a potential source of variance for example. One study partly used an HMD and partly a CAVE system as the VR presentation mode (see Table 2), but in this case no significant differences in the outcome measures were found (Meyerbroeker et al., 2013).

Concerning the treatment procedure, a similar amount of exposure in the VR and *in vivo* condition was required according to the eligibility criteria. Nevertheless, the exposure environment in VR and *in vivo* was not always equal (see Table 2). For example, two studies conducted a flight exposure in VR, but only exposed participants to sitting on a stationary plane with imaginal exposure of a flight in the *in vivo* condition (Rothbaum et al., 2000, 2006). Moreover, over different studies there were differences in the amount and kind of therapeutic techniques applied for pre- and post-proceeding and to accompany exposure treatment (see Table 2). For example, Social Phobia exposure was combined with cognitive techniques in the study by Bouchard et al. (2016), but not in the study by Kampmann et al. (2016).

A limitation of the included data is that the original studies differed in providing either data on an intent-to-treat sample, a completer sample, or both. If available, intent-to-treat data was used for synthesis, but if not provided, data on completer samples was included (see Table 4). This should be considered as a potential source of bias. Future original studies should thus provide data on both samples, so that separate meta-analysis can be conducted.

A limitation concerning the synthesis of data is the use of fixed-effect models for pooling a smaller number of studies on one specific kind of phobic anxiety disorder. A fixed-effect model requires functionally identical studies (Borenstein et al., 2009), which can approximately be reached by the strict inclusion criteria and focusing on only one phobic disorder for the synthesis. Nevertheless, the included studies on one phobic anxiety disorder were not entirely identical and therefore the results must be interpreted cautiously and should not be used for generalizations on a wider population (Borenstein et al., 2009). They do however provide a descriptive analysis of differences concerning the treatment effects of VR and *in vivo* exposure therapy in different phobias and allow a discussion on potential mechanisms behind differences in effect sizes for the comparison of VR and *in vivo* exposure therapy between different phobic anxiety disorder.

Furthermore, the applied statistical tests are only valid for testing differences between groups, but not for proving equivalency. Therefore, the non-significant results have to be interpreted cautiously and the relevance of effect sizes has to be considered. Future meta-analyses based on a larger number of trials should also draw on non-inferiority or equivalence tests (Piaggio et al., 2006) to examine the equivalence of VR and *in vivo* exposure.

Finally, we could not conduct a statistical analysis of potential effective factors of VR exposure therapy due to the small number of available studies. As soon as more original studies have been published, this will also be an important research question for future meta-analyses.

In general, this meta-analysis and the original studies were conducted by researchers in the field of VR. This is a potential source of bias, especially as no pre-registration of this systematic review and meta-analysis protocol was conducted. However, we comprehensively disclose our methods and results. Furthermore, as there are no original studies available from field-independent researchers, we provide a comprehensive and objective description of the materials and procedures of all studies included in this meta-analysis. In this regard, we want to enable the reader to capture information for an independent assessment and interpretation of the results and thereby attenuate the bias caused by non-independent researchers.

### Conclusions

This meta-analysis provides results on a head to head comparison of VR exposure and *in vivo* exposure as the golden standard of treatment for phobias, synthesized from methodologically comparable studies with an equivalent amount of exposure in VR and *in vivo*, and with equivalent interventions applied alongside VR and *in vivo* exposure in phobic anxiety disorders, especially in Agoraphobia and Specific Phobia. For Social Phobia, the synthesized effect size points to a superiority of *in vivo* exposure, but the wide range of effect sizes for the individual studies also shows the high potential of VR exposure in this phobic anxiety disorder.

While the individual effect sizes for the studies on Agoraphobia both indicate equivalency between VR and *in vivo* exposure, the individual effect sizes for the studies on Specific Phobia and Social Phobia ranged from inferiority to equivalency and even superiority of VR exposure. Studies that yielded an equivalent or even superior effect of VR exposure combined an exposure of agoraphobic situations in VR and *in vivo* with cognitive interventions and interoceptive exposure (Botella et al., 2007; Meyerbroeker et al., 2013), realized an equivalent environment for the exposure of fear of heights in VR as *in vivo* and allowed patients to move in VR by walking around freely in a demarcated space (Emmelkamp et al., 2002), or focused on reinterpretations without requiring habituation during VR and *in vivo* exposure in Social Phobia and applied social interactions realizing rejection experiences in the VR condition (Bouchard et al., 2016). Although a statistical analysis of potential effective factors was not possible, such observations can contribute to the implementation of maximized efficacious VR exposure therapy. There are hints that VR exposure in Social Phobia should be combined with cognitive interventions and should use the possibility to manipulate virtual agents in order to target central fears of Social Phobic patients to reach equal or even better efficacy in comparison to *in vivo* exposure.

Considering the results of this meta-analysis, and because there are barriers in conducting *in vivo* exposure in clinical practice (Neudeck and Einsle, 2012), it would be strategically useful to promote the dissemination of VR comprehensively. As VR therapy is time-effective, accounts for less organizational effort (Diemer et al., 2015), and has a higher acceptance in patients (García-Palacios et al., 2007), this could be a feasible possibility in achieving efficacious exposure treatment for a wider population of patients.

As there were only a few studies with sufficiently homogenous materials and procedures published on the examined research question, this meta-analysis is still based on a small number of studies. A proportionally high level of internal validity can be expected for the results, but the generalizability should be verified in an updated meta-analysis as soon as more studies are published in the following years. In addition, future research should focus on the effective factors of VR exposure therapy and further examine mechanisms enhancing the treatment effects that may be applicable using VR exposure. Examples are the impact of cognitive strategies (see the study conducted by Bouchard et al., 2016), the movement mode in VR (mouse/gamepad vs. walking freely in a demarcated space as achieved in the study conducted by Emmelkamp et al., 2002), or the repetition of exposure with different stimuli or contexts (Shiban et al., 2013, 2015). Further interesting research questions include multimodal exposure (Peperkorn et al., 2016), eligible patient population, or a different amount of VR sessions applied. As VR materials and procedures continuously improve, superior effects of VR exposure in comparison to *in vivo* exposure therapy could be realized in future. This is because VR has the possibility to create ideal environments for exposure, for example virtual rejection experiences targeting central fears of Social Phobia patients as for example achieved in the study conducted by Bouchard et al. (2016), and also has the possibility to consider and test theoretical and practical concepts, for example inhibitory learning and inhibitory regulation (Craske et al., 2008; Craske, 2015). The creation of complex virtual social interactions is therefore a challenge that can be solved in future research and VR scenario development.

## Author Contributions

AM and TW developed the idea of the research question. FK performed the systematic literature search to identify published studies, and conducted the title, abstract and full-text screening. TW also conducted abstract and full-text screening, selecting eligible studies. AM was consulted to discuss disagreements during the screening process. TW and FK performed the data extraction. TW conducted the statistical analyses with contributions from FK and AM. TW and AM interpreted the findings and wrote the manuscript draft.

### Conflict of Interest Statement

AM is stakeholder of a commercial company that develops virtual environment research systems. The remaining authors declare that the research was conducted in the absence of any commercial or financial relationships that could be construed as a potential conflict of interest.
